# Supplementary material for: A single‐cell RNA labeling strategy for measuring stress response upon tissue dissociation
Source: Mol Syst Biol. 2022 Dec 27;19(2):e11147. doi: 10.15252/msb.202211147 (PMC9912023; doi:10.15252/msb.202211147)
Supplement: Supplementary file 1 — Expanded View Figures PDF [file MSB-19-e11147-s001.pdf]

# Expanded View Figures

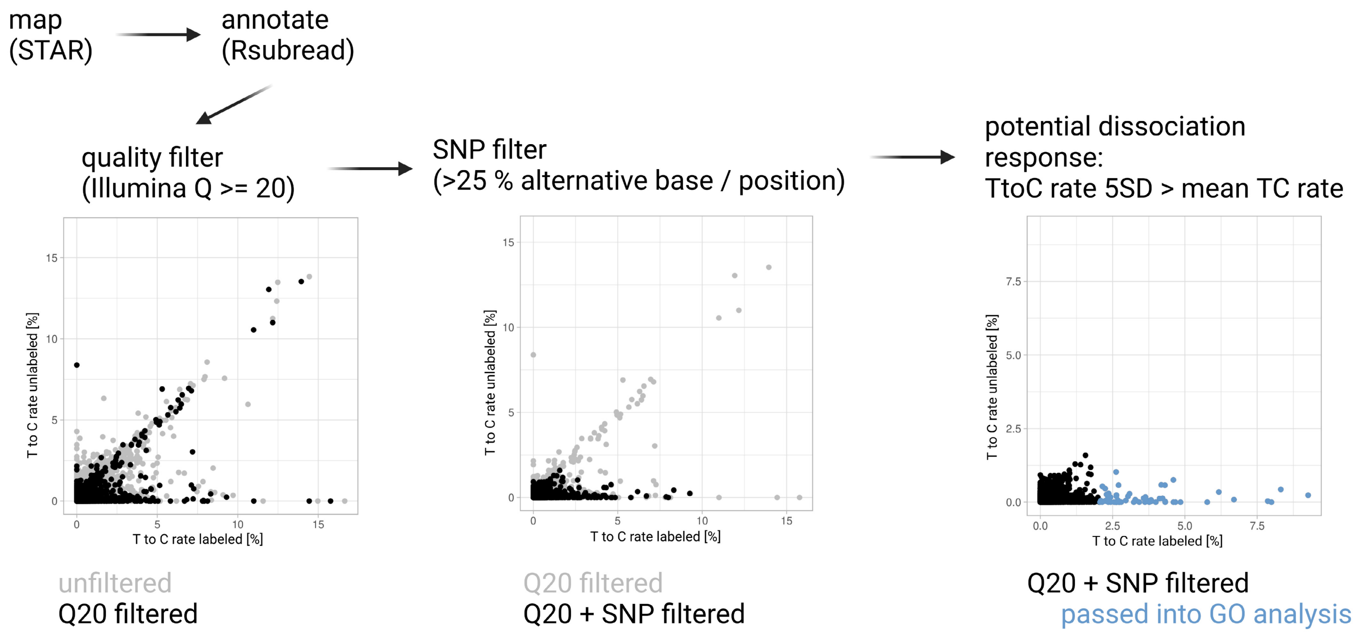


**Figure EV1. Computational workflow for bulk data.**

Reads are mapped using STAR, annotated using Rsubread and passed through quality and SNP filtering steps before selecting genes for GO analysis based on a T-to-C conversion rate at least 5 standard deviations larger than the mean.


© 2022 The Authors

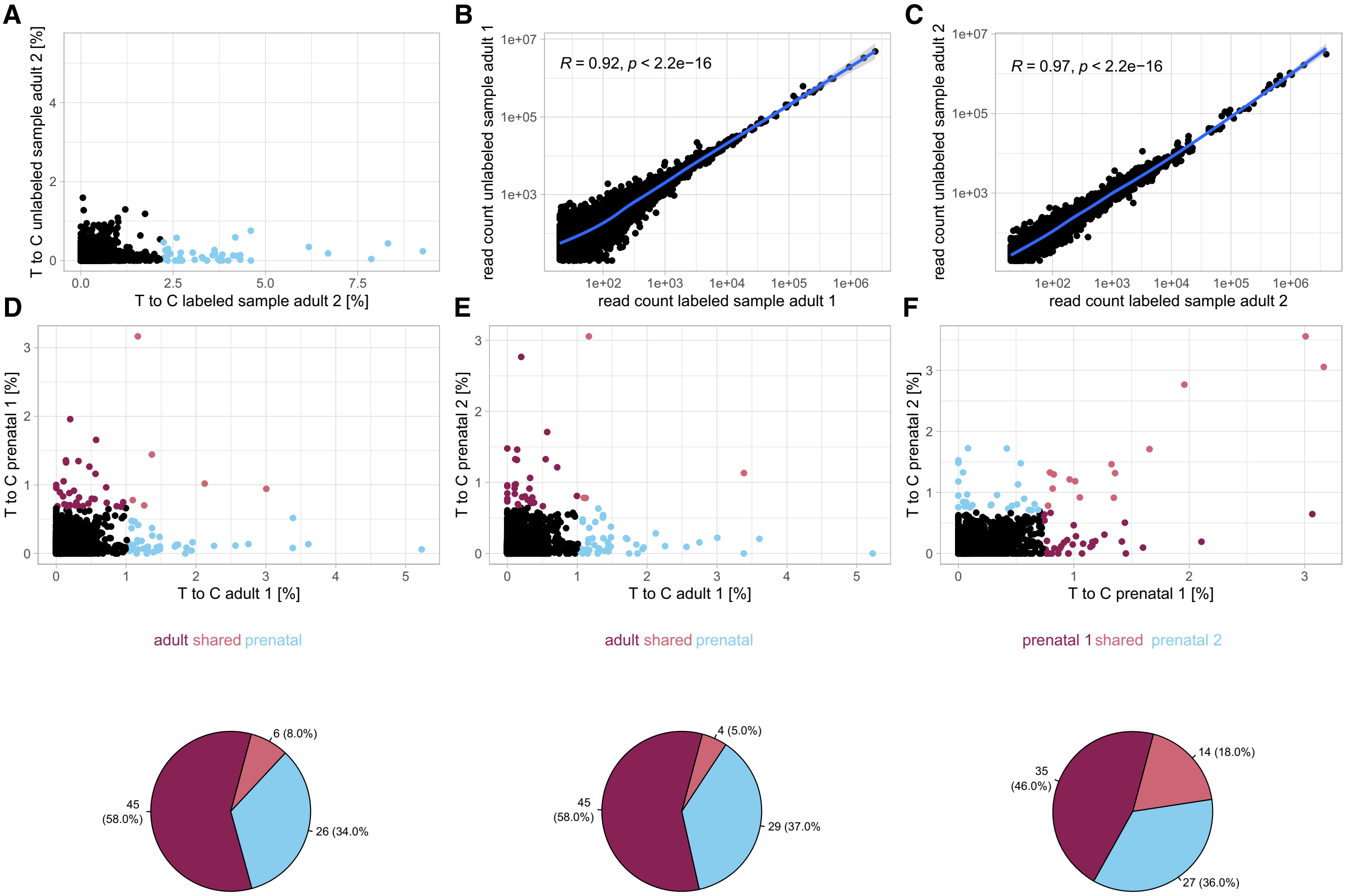


**Figure EV2. Biological replicates for cardiomyocyte dissociation experiments.**

A Adult mouse cardiomyocytes, replicate 2, T-to-C rates per gene in labeled (4sU added) and unlabeled (no 4sU added) sample. Blue (~ 1% of genes): T-to-C rate ≥ 5 standard deviations.

B, C Read count correlation between labeled and unlabeled adult mouse cardiomyocyte samples. Blue lines show sliding window average (smoothed conditional mean) and 95% confidence interval. *R* values calculated through Pearson correlation.

D, E T-to-C rates per gene in prenatal vs. adult cardiomyocytes. Genes with T-to-C rate ≥ 5 standard deviations in one or both samples are highlighted.

F T-to-C rates per gene in prenatal replicate 1 vs. prenatal replicate 2. Genes with T-to-C rate ≥ 5 standard deviations in one or both samples are highlighted.

© 2023 The Authors

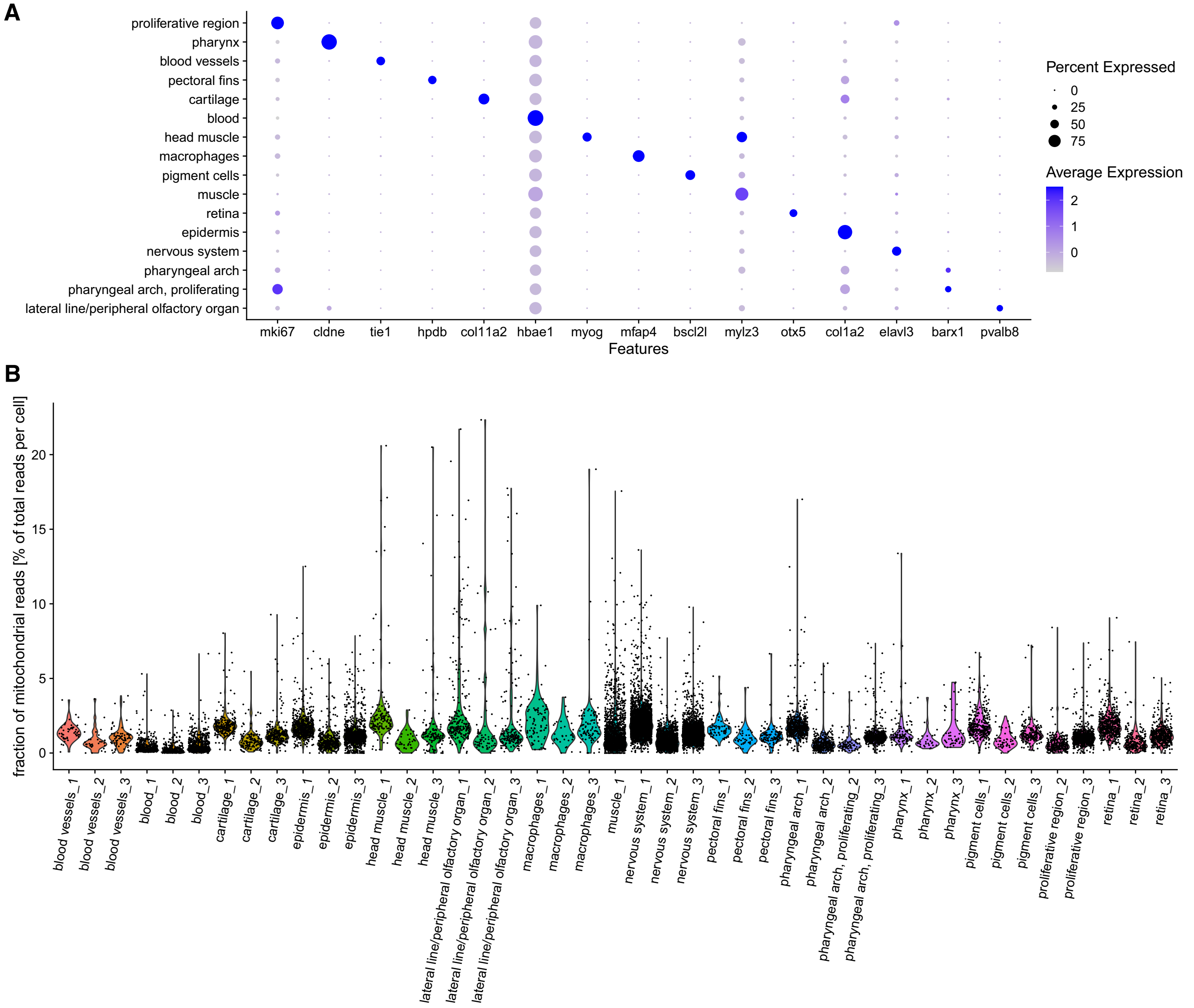


**Figure EV3. Characterization of single cell zebrafish data.**

A Marker gene expression in 48 hpf zebrafish cells, data from all three samples is merged.

B Fraction of mitochondrial reads per cell, cell types are separated by sample of origin.

 © 2022 The Authors

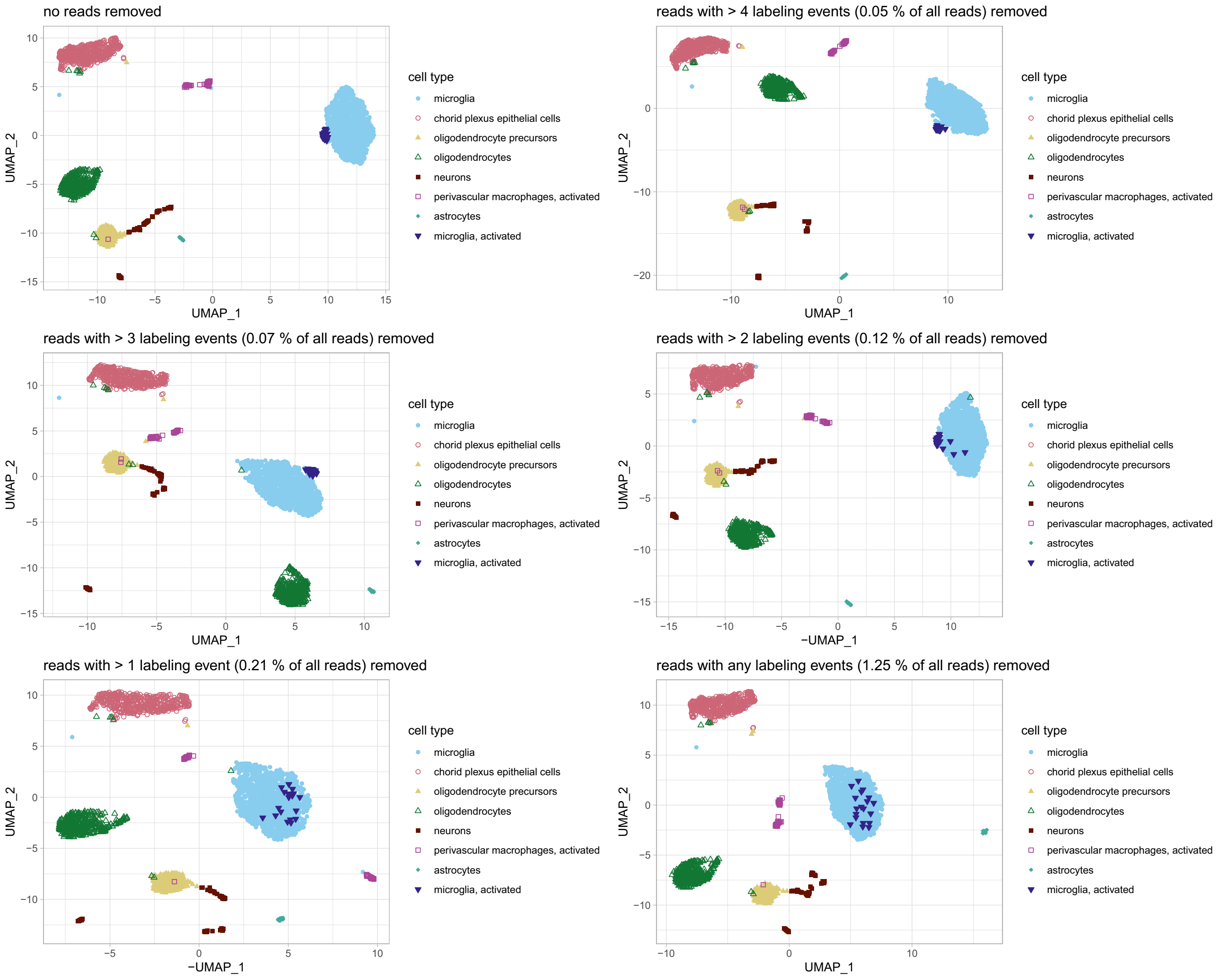


**Figure EV4. Dependence of clustering results on labeled transcripts.**

Gradual removal of labeled UMIs leads to the activated microglia cluster merging with the non-activated microglia.
