# Supplementary material for: A single‐cell RNA labeling strategy for measuring stress response upon tissue dissociation
Source: Mol Syst Biol. 2022 Dec 27;19(2):e11147. doi: 10.15252/msb.202211147 (PMC9912023; doi:10.15252/msb.202211147)
Supplement: Supplementary file 7 — PDF+ [file MSB-19-e11147-s004.pdf]

Method

TRANSPARENT PROCESS OPEN ACCESS

molecular systems biology

A single-cell RNA labeling strategy for measuring stress response upon tissue dissociation

Anika Neuschulz[1,2], Olga Bakina[3], Victor Badillo-Lisakowski[2,4,5], Pedro Olivares-Chauvet[1], Thomas Conrad[6], Michael Gotthardt[4,5,7], Helmut Kettenmann[3] & Jan Philipp Junker[1,5,7,*]

Abstract

Tissue dissociation, a crucial step in single-cell sample preparation, can alter the transcriptional state of a sample through the intrinsic cellular stress response. Here we demonstrate a general approach for measuring transcriptional response during sample preparation. In our method, transcripts made during dissociation are labeled for later identification upon sequencing. We found general as well as cell-type-specific dissociation response programs in zebrafish larvae, and we observed sample-to-sample variation in the dissociation response of mouse cardiomyocytes despite well-controlled experimental conditions. Finally, we showed that dissociation of the mouse hippocampus can lead to the artificial activation of microglia. In summary, our approach facilitates experimental optimization of dissociation procedures as well as computational removal of transcriptional perturbation response.

Keywords cellular stress response; RNA labeling; single-cell transcriptomics; SLAM-seq; tissue dissociation
Subject Categories Chromatin, Transcription & Genomics; Methods & Resources
DOI 10.15252/msb.202211147 | Received 29 May 2022 | Revised 12 December 2022 | Accepted 13 December 2022 | Published online 27 December 2022
Mol Syst Biol. (2023) 19: e11147

Introduction

Single-cell transcriptomics has emerged as the method of choice for systematic identification of cell types or states in complex tissues. However, tissues typically need to be dissociated into a single-cell suspension for analysis, which triggers a transcriptional response that can confound the downstream analysis (Denisenko *et al*, 2020; Mattei *et al*, 2020). This is particularly problematic when investigating biological perturbations that may resemble dissociation response (e.g. reaction to tissue injury), when comparing conditions that may be affected differentially by dissociation (e.g. embryonic versus adult tissue), or when analyzing tissues that require harsh dissociation conditions. Dissociation response may lead to batch differences and can also produce artificial transcriptional diversity within a cell type, as was shown for a stress-induced sub-population of stem cells (van den Brink *et al*, 2017). Dissociation protocols, therefore, need to be carefully optimized when performing high-resolution analysis of cellular activation states (Hrvatin *et al*, 2018). General transcription inhibitors have been used for minimizing the influence of tissue dissociation on gene expression measurements (Wu *et al*, 2017), but such treatments can lead to cell death (Bensaude, 2011) and may create biases in the detected gene expression (Cassé *et al*, 1999). Analysis of single nuclei instead of single cells allows for faster sample preparation and can minimize transcriptional stress response as well as other biases (Lacar *et al*, 2016) at the expense of reduced data quality with respect to the number of transcripts and genes detected per cell. It, therefore, remains an important open challenge to directly measure transcriptional response to dissociation. Such an approach could enable systematic experimental optimization of dissociation conditions and allow computational removal of transcriptional dissociation response.

Results

We reasoned that the scSLAM-seq method for RNA labeling (Erhard *et al*, 2019; Cao *et al*, 2020; Holler *et al*, 2021), which is based on the incorporation of ribonucleotide analogs into newly transcribed RNA, can be repurposed for measuring dissociation response. Adding the uridine analog 4-thiouridine (4sU) to the dissociation reaction should label the transcripts that are synthesized during the dissociation procedure (Fig 1A). After a thiol modification step using iodoacetamide, labeled transcripts can be identified in the sequenced data by characteristic T-to-C substitutions (see "Step by Step protocol", in the Materials and Methods). Using cultured mouse embryonic stem cells (mESC), we confirmed that our

1 Quantitative Developmental Biology, Berlin Institute for Medical Systems Biology, Max Delbrück Center for Molecular Medicine, Berlin, Germany
2 Humboldt-Universität zu Berlin, Berlin, Germany
3 Cellular Neurosciences, Max Delbrück Center for Molecular Medicine, Berlin, Germany
4 Neuromuscular and Cardiovascular Cell Biology, Max Delbrück Center for Molecular Medicine, Berlin, Germany
5 DZHK (German Centre for Cardiovascular Research), Partner Site Berlin, Berlin, Germany
6 BIH/MDC Genomics Technology Platform, Berlin, Germany
7 Charité Universitätsmedizin Berlin, Berlin, Germany
*Corresponding author. Tel: +49 30 9406 1860; E-mail: janphilipp.junker@mdc-berlin.de

© 2022 The Authors. Published under the terms of the CC BY 4.0 license.

workflow specifically increased the rate of T-to-C transitions (Fig 1B). Typical 4sU concentrations are in the range of 100 μM. However, in our cell culture experiments we found that concentrations of up to 10 mM were possible, even for an extended labeling period of 25 h. In the following, we therefore used a concentration of 10 mM 4sU, in order to enable high labeling rates despite the short time window of tissue dissociation and the difficulty of reaching high intracellular 4sU concentrations in the initially multilayered tissue. However, we wish to note that we do not recommend such high concentrations for applications requiring longer labeling times. In general, we would suggest checking the effect of 4sU on transcription for any RNA labeling experiment.

As a first experiment to measure dissociation response in bulk, we compared zebrafish larvae (48 h post fertilization, hpf) treated with 4sU during dissociation to untreated larvae that were homogenized for analysis (Fig 1C). To minimize false positive labeling events (i.e., "labeled" genes in the untreated sample), we required a minimum sequencing quality of Q20 (base call accuracy of 99%) to call a T-to-C substitution, and we filtered against SNPs (single nucleotide polymorphisms) by removing all T-to-C transitions that occurred at a frequency > 25% (Fig EV1). This approach effectively removed false positives and allowed identification of genes that are transcribed during the dissociation procedure based on their high T-to-C transition rate (Fig 1C).

We considered that genes might be detected as highly labeled due to two different reasons: Either these genes are strongly upregulated upon dissociation and constitute a *bona fide* stress response; or, alternatively, the detected genes are expressed constitutively and are not related to stress response, but are merely characterized by a particularly high turnover rate. To distinguish between these two scenarios, we compared zebrafish larvae that were labeled during dissociation to larvae that were labeled *in vivo* via microinjection of 4sU for the same amount of time (30 min) and homogenized for analysis (Fig 1D, Dataset EV1). We found that only 12% of genes (or 11 out of the 95 highly labeled genes) showed high labeling in both conditions, suggesting that genes with high turnover rates are typically not constitutively expressed. The category of high turnover genes mainly consisted of developmental regulators including members of the *her* family and other transcriptional regulators. By contrast, we found a larger set of genes that were labeled more highly in the dissociated compared to the injected and homogenized larvae, and which contained typical stress response genes like *fos/jun* and heat shock genes. Interestingly, we also found a different set of genes that were labeled more highly in the injected larvae. We speculate that such genes might be related to stress response due to injection or might reflect minor differences in staging. After the successful characterization of the approach, we decided to use our method to compare two different protocols for preparation of single-

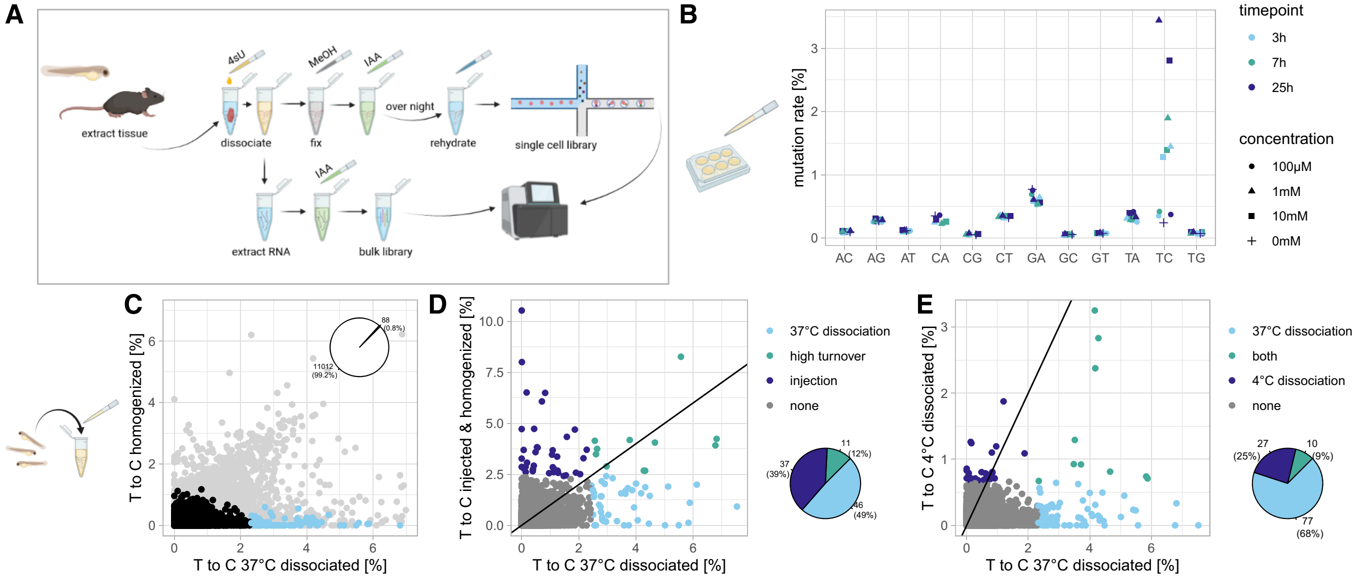

Figure 1. Measuring dissociation response by RNA labeling.

A Experimental workflow for labeling transcripts during dissociation. 4sU is added to the dissociation buffer. For single-cell sequencing, cells are fixed in methanol and a iodoacetamide (IAA) treatment is carried out over night before rehydrating the cells and passing them into the 10× chromium workflow. For bulk sequencing, the IAA treatment is carried out on extracted RNA, followed by library preparation. See Materials and Methods for detailed protocol.

B Bulk mutation rates in cultured mouse embryonic stem cells after 4sU exposure.

C T-to-C rates per gene in unlabeled and homogenized vs. labeled and dissociated (30 min, 37°C) 48 hpf zebrafish larvae. Genes with a T-to-C rate ≥ 5 standard deviations (SD) are highlighted in blue (~ 1% of genes). For comparison, light gray data points show T-to-C rates without SNP removal, which include many false positives in the unlabeled dataset.

D T-to-C rates per gene in 4sU injected (30 min incubation) and homogenized 48 hpf zebrafish larvae vs labeled and dissociated (30 min, 37°C) larvae. Genes with T-to-C rate ≥ 5 SD in one or both samples are highlighted.

E T-to-C rates per gene in cold dissociated (30 min, 4°C) 48 hpf zebrafish larvae vs warm dissociated (30 min, 37°C) larvae. Genes with T-to-C rate ≥ 5 SD in one or both samples are highlighted.

 © 2022 The Authors

cell suspensions: a standard dissociation at 37°C and a cold dissociation at 4°C. As expected, we found a lower degree of labeling upon cold dissociation, which might reflect more gentle dissociation conditions as well as overall reduction of gene expression activity at 4°C (Fig 1E). However, cold dissociation still led to expression of heatshock genes, and we also observed upregulation of a cold dissociation-specific gene set (Dataset EV1).

Next, we applied our approach to adult mouse cardiomyocytes, which require more extensive dissociation procedures involving isolation of cells after perfusion of the extracted heart (Rudolph *et al*, 2019; Materials and Methods), and might therefore be affected by stronger dissociation response. Similar to zebrafish larvae, we found that RNA labeling during dissociation led to identification of a small set of genes (~ 0.8% or 44 out of 5,244 detected genes) with high labeling rates (Figs 2A and EV2). Importantly, addition of 4sU during dissociation did not lead to detectable gene expression changes compared to dissociation without 4sU, suggesting that the 4sU treatment does not create gene expression artifacts (Fig EV2B and C). We decided to use this system to assess the reproducibility of dissociation response by comparing five samples of adult cardiomyocytes. While four of these samples were processed under identical conditions, we increased the temperature during dissociation from 37°C to 42°C in one sample (adult 5) for comparison. Additionally, we added three replicates of prenatal cardiomyocytes, which can be isolated with a milder dissociation procedure (Materials and Methods), and which we hypothesized to show a lower level of dissociation response. We noticed that, in addition to a shared set of labeled genes (including genes involved in metabolic and regulatory processes regarding cell death and response to stress), the replicates also showed distinct sample-specific transcriptional profiles (Fig 2B–D, Dataset EV2). In particular, sample adult 5 (42°C dissociation) displayed a more pronounced stress response, including GO terms related to unfolded protein response (Dataset EV2). This suggests that, at least in experimentally challenging systems like the heart, minor differences during the dissociation procedure can result in qualitatively different perturbation responses, which may potentially manifest as batch effects in single-cell RNA-seq datasets. However, we could also identify a core set of 17 genes that were strongly labeled in at least three of the five adult samples, including some genes known to be involved in stress response (*Fos/Jun*, *Atf3*, *Gadd45g*), members of the actin family and extracellular matrix components, as well as five so far unclassified genes (Dataset EV3). In comparison, the prenatal cardiomyocytes showed a weaker dissociation response, with a high degree of similarity among the replicates (Fig 2C and D). In summary, we conclude that RNA labeling during tissue dissociation is an effective method for identifying genes whose expression may be dominated by stress response. While we were able to determine a core set of dissociation response genes in adult cardiomyocytes, we also detected a substantial amount of sample-to-sample variation. Furthermore, we observed differences in dissociation response between prenatal and adult cardiomyocytes, which may be problematic when performing comparisons between these conditions without correcting for dissociation response.

After the bulk analysis of zebrafish larvae and mouse cardiomyocytes, we next wanted to investigate dissociation response at single-cell level. For this analysis we again chose to work with zebrafish larvae at 48 hpf, comparing three conditions for

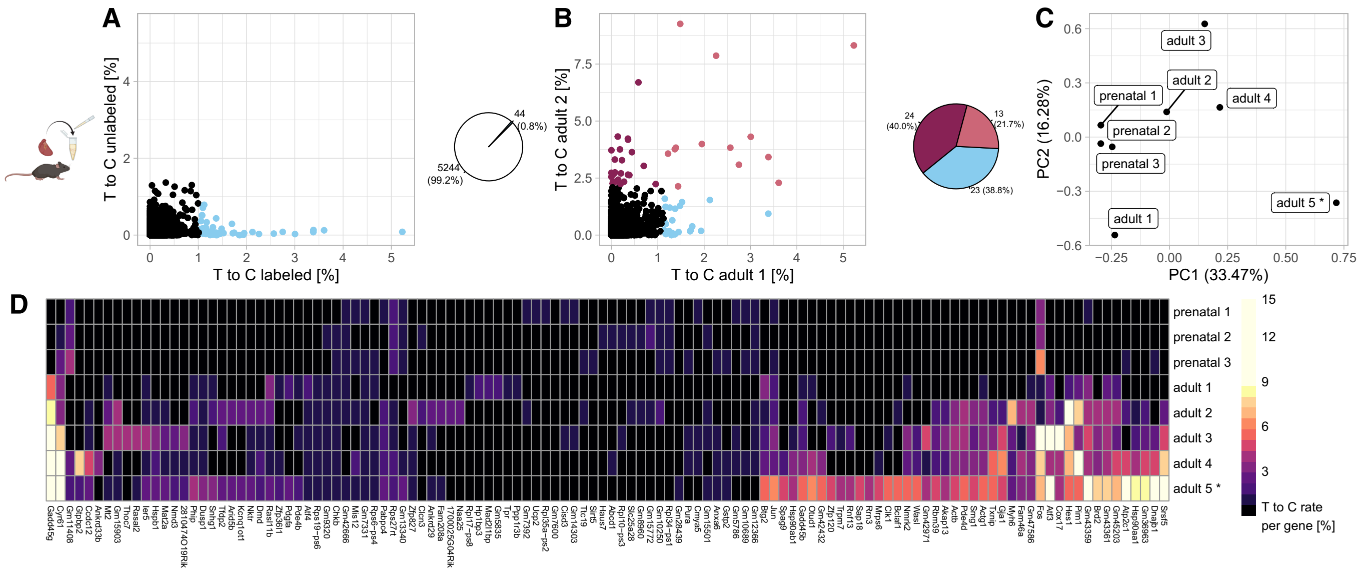

Figure 2. Variation of dissociation response in similar samples.

A T-to-C rates per gene in labeled (4sU added) and unlabeled (no 4sU added) adult mouse cardiomyocytes. Blue (~ 1% of genes): T-to-C rate ≥ 5 SD.

B Comparison of the labeled sample in (A) to a biological replicate. Genes with T-to-C rate ≥ 5 SD in one or both samples are highlighted.

C Principal component analysis of dissociation response in prenatal and adult cardiomyocytes. PCA was calculated based on T-to-C rates of all genes which had a T-to-C rate >= 1 SD of the mean in at least one sample. Sample adult 5 was dissociated at 42°C, all others at 37°C.

D Heatmap showing consensus and sample specific dissociation response. Genes shown have a T-to-C rate >= 5 SD of the mean in at least one sample. Sample adult 5 was dissociated at 42°C, all others at 37°C.

© 2022 The Authors

preparation of a single-cell suspension (Materials and Methods): 30 min dissociation; 30 min dissociation followed by FACS (which we here used to sort for cells of expected size and granularity; 10 min sort plus 20 min of setup and washes); and finally 30 min dissociation and a similar FACS procedure, followed by 30 min on ice (to simulate waiting times that arise when preparing multiple samples). To analyze RNA labeling on the single-cell level, we fixed the dissociated and 4sU-treated cells in methanol, followed by iodoacetamide treatment in intact cells (Holler *et al*, 2021) and library preparation using the 10× Genomics Chromium system (Materials and Methods). In total we sequenced ~ 25,200 cells, which computationally clustered into 16 main cell types (Figs 3A and EV3, Materials and Methods). Comparing the three dissociation conditions, we observed overall low batch effects (Fig 3B), with the exception of neuronal cells, which showed higher levels of mitochondrial reads in the non-FACSed samples, suggesting a depletion

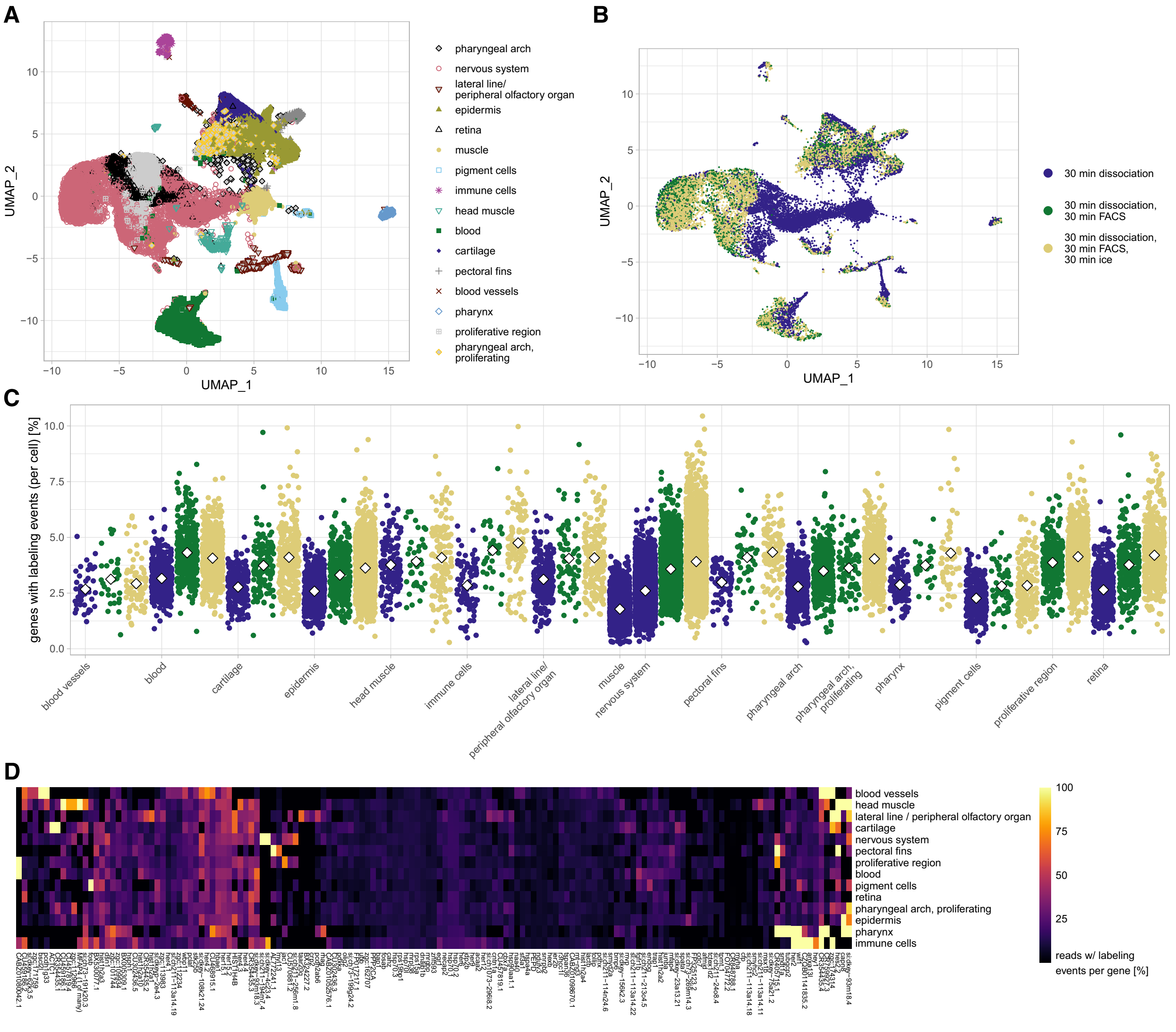

Figure 3. Shared and cell type specific dissociation response.

A Clustering of 48 hpf zebrafish larvae cells dissociated at 37°C for 30 min and either fixed immediately or FACS sorted and incubated further on ice (see Materials and Methods for details).

B Sample of origin for the clustering shown in (A).

C Percentage of genes with labeling events for each cell shown in (A) and (B). White diamonds indicate mean values. Not all cell types could be found in every sample (e.g. muscle cells were depleted in FACS samples, see main text).

D Heatmap showing cell type specific and core dissociation response in 48 hpf zebrafish larvae cells dissociated (30 min, 37°C), FACS sorted and incubated on ice for 30 min. Genes shown have at least 250 reads with >= 10% of those containing >= 2 labeling events in at least one cell type.

 © 2022 The Authors

of low quality cells through FACS. Furthermore, muscle cells were depleted in the FACSed samples, which again suggests that these cells were lost, possibly due to their larger size (Fig EV3). Analysis of T-to-C rates showed higher labeling of the FACSed samples, in line with their longer incubation time after exposure to 4sU as well as differences in labeling rate between the detected cell types (Fig 3C). Comparison of the most highly labeled genes (> 10% of UMIs with ≥ 2 labeling events) revealed a shared dissociation program across cell types including genes such as *endog*, *pclaf*, members of the heat shock family and *fos/jun* family, as well as cell type specific programs (Fig 3D, Dataset EV4). In summary, we demonstrate that single-cell RNA labeling can be used to measure dissociation response in a sample and cell type specific manner.

As a second test case for single-cell analysis of dissociation response, we chose to work with mouse microglia, which are known to have important functions in brain homeostasis and disease (Keren-Shaul *et al*, 2017; Hammond *et al*, 2019; Olah *et al*, 2020). Microglia exhibit great transcriptomic variety (Zeisel *et al*, 2018; Ochocka *et al*, 2021) and have been demonstrated to react upon enzymatic digestion (Mattei *et al*, 2020; Marsh *et al*, 2022). We decided to focus on microglia contained in the mouse hippocampus, a brain region that allows for consistent dissection (Fig 4A). We dissociated the samples using a mild protocol optimized for gentle dissociation of brain tissue and subsequent isolation of microglia (Materials and Methods). Clustering analysis revealed the expected cell types, including microglia as well as a subgroup of activated microglia (Fig 4B and C). Given the known sensitivity of microglia to changes in their environment, we wondered whether the sub-cluster of activated microglia characterized by *fos/jun* expression might be an experimental artifact caused by tissue dissociation. Indeed, we found that the activated microglia fully merged with the main microglia cluster when removing all

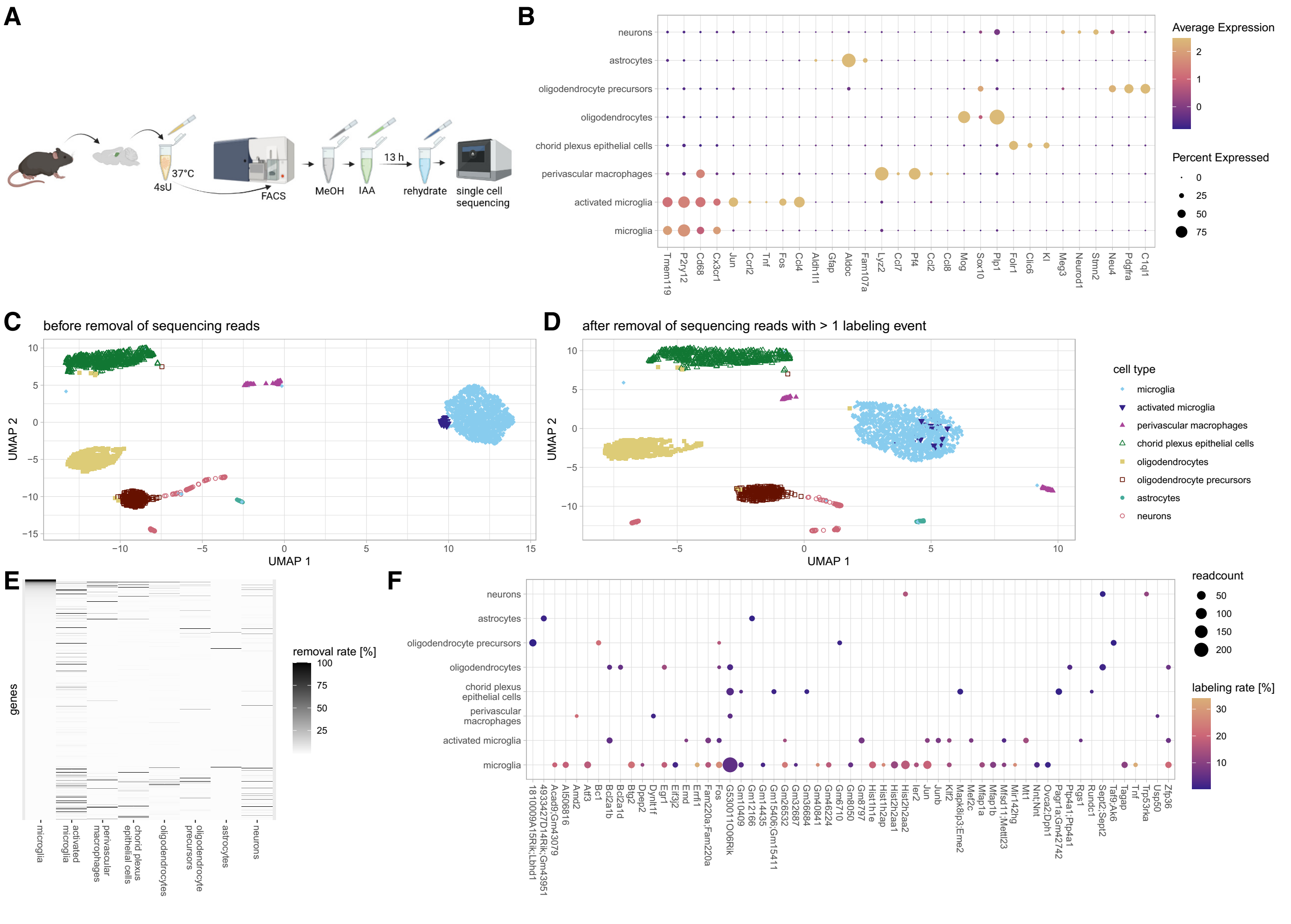

Figure 4. Single-cell analysis of dissociation response in the mouse hippocampus.

A Experimental workflow for RNA labeling in hippocampus cells during dissociation. Hippocampi are extracted, dissociated in the presence of 4sU (30 min, 37°C) and sorted for live cells. Sorted cells are fixed in methanol before IAA treatment and rehydration, followed by the standard 10× genomics workflow.

B Marker gene expression in sorted mouse hippocampus cells.

C, D Clustering of sorted mouse hippocampus cells before (C) and after (D) removal of UMIs with 2 or more labeling events.

E Removal rates of reads per gene and cell type.

F Labeling rates and counts of removed reads. Genes with at least 5% of reads (min 3 reads) removed are shown.

transcripts with ≥ 2 T-to-C conversions from the dataset (Figs 4C and D, and EV4). Of note, in this analysis we only removed individual labeled transcripts, but we retained all transcripts of the corresponding genes with < 2T-to-C conversions, so no genes were excluded from further analysis. This observation clearly demonstrates the ability of our approach to separate dissociation response from real biological effects, thereby increasing the resolution of single-cell RNA-seq analysis. While the overall fraction of labeled transcripts was low (< 1%), for a small group of genes > 50% of reads were removed as putative dissociation response (Fig 4E). Transcriptional dissociation response varied between different cell types and was strongest in microglia (Fig 4F). We applied GO term analysis to genes for which ≥ 3 transcripts with ≥ 2 labeling events were removed and observed ~ 90% of those genes contributing to stress/death related GO terms in activated microglia. For non-activated microglia ~ 45% fell into this category. None of the other cell populations had a considerable portion of stress or death related GO terms (Dataset EV5).

Discussion

In summary, our analysis revealed that SLAM-seq can be used for measuring dissociation response, a major confounding factor in single-cell RNA-seq analysis. Our combined experimental and computational approaches allow identification and removal of transcripts that are made during tissue dissociation, which can help identify experimental artifacts and increase the resolution of subsequent analysis. Here we used analysis on the bulk and single-cell level for measuring dissociation response. Our scSLAM-seq protocol is based on the 10× Genomics Chromium system and requires methanol fixation of cells prior to iodoacetamide treatment. While we did not observe any reduction of data quality following methanol treatment, we wish to note that there are other methods for preparation of scSLAM-seq libraries that do not require fixation (Erhard *et al*, 2019; Hendriks *et al*, 2019; Qiu *et al*, 2020).

Using SLAM-seq for measuring dissociation response may be particularly useful for cell types that are suspected to produce dissociation artifacts, either because harsh dissociation conditions are required (e.g. cardiomyocytes) or because the cells are particularly responsive to perturbation (e.g. microglia). Our analysis identified a core set of genes that should be considered as problematic because they consistently appear in the dissociation response of different cell types and systems. However, in addition to this generic dissociation response signature, we observed that different cell types and individual samples can also react in distinct ways to dissociation. For microglia from the mouse hippocampus, we demonstrated that activation response caused by tissue dissociation can be computationally corrected by removing labeled transcripts from the analysis. While it should be possible to regress out dissociation response from scRNA-seq datasets based on our list of core dissociation genes without performing additional SLAM-seq experiments, akin to removal of cell cycle effects in scRNA-seq, we wish to note that this can be potentially problematic, since cellular stress response may not only be due to dissociation but can also be caused by biological factors. Activated microglia are an example for this scenario: While we found microglia activation to be an artifact created by tissue dissociation in Fig 4, microglia activation can also arise disease contexts. By regressing out dissociation-related programs, one might therefore also inadvertently remove a *bona fide* biological response from the tissue sample. In contrast to dissociation response, the cell cycle is a well-defined gene expression program that is strongly separated from other cellular programs (Schwabe *et al*, 2020); hence, no such concerns apply to computational removal of cell cycle effects.

Materials and Methods

Reagents and Tools table

Reagent / resource	Reference / source	Identifier /catalog number
**Experimental models**		
CD1 feeder cells	charles river	CD-1® IGS Mouse
G4 ES cells	George *et al* (2007)	RRID:CVCL_E222
C57BL/6N	Jackson Laboratories	
**General Reagents**		
4sU	Merck Chemicals GmbH	#T4509
TRIzol	Life Technologies GmbH	#15596018
IAA	Merck Chemicals GmbH	#11149
**Cell culture Reagents**		
DMEM	Gibco	#10829-018
FCS	Biochrom	#S0615
Glutamine	Lonza	#BE17-605E
Penicillin/streptomycin	Lonza	#DE17-603
Nonessential amino acids	Gibco	#11140-35

© 2022 The Authors

Reagents and Tools table (continued)

Reagent / resource	Reference / source	Identifier /catalog number
10 mM β-mercaptoethanol	Sigma	#M-7522
Nucleosides	Chemicon	#ES-008D
Murine Leukemia Inhibitory Factor	Chemicon	#ESG1107
PBS	Lonza	#BE17-512F
Trypsin	Gibco	#25300-054
**Zebrafish experiments – Reagents**		
Dextran fluorescein	Thermo Fisher Scientific	#7136
HBSS	Life Technologies GmbH	#14175095
TrypIE	Life Technologies GmbH	#A1217701
Native Bacillus licheniformis Protease	Creative Enzymes	#NATE-0633
EDTA	Molekula	#900493
BSA	Sigma-Aldrich	#A4503
DPBS	Gibco	#14190144
**Adult mouse cardiomyocytes – Reagents**		
Stock perfusion buffer	Rudolph *et al* (2019)	
2,3-Butanedionemonoxime	Rudolph *et al* (2019)	
Glucose	Sigma-Aldrich	#G7528
Perfusion buffer pH 7.46	Rudolph *et al* (2019)	
Liberase blendzyme 1	Roche	#1988417
Trypsin	Gibco/Invitrogen	#15090046
Bovine calf serum	HyClone	#SH30073
**Embryonic mouse cardiomyocytes – Reagents**		
Trypsin-EDTA	Thermo Fisher Scientific	#25200056
Reconstituted fluorescent reactive dye	Thermo Fisher Scientific	#L34955
Bovine calf serum	HyClone	#SH30073
Alexa Fluor 647 Rat Anti-Mouse VCAM-1	BD Biosciences	#561612
**Mouse hippocampus – Reagents**		
Adult Brain Dissociation Kit	Miltenyi Biotec	#130-107-677
Narcorene	Merial GmbH	
Heparin-Natrium-250,000-ratiopharm	Ratiopharm	
Calcein AM dye	BD Pharmingen	#564061
SSC	Carl Roth GmbH	#1054.1
BSA	Sigma-Aldrich	#A4503
SuperRNase IN	Life Technologies GmbH	#AM2694
DTT	Carl Roth GmbH	#6908.1
**Bulk RNA extraction**		
GlycoBlue	Life Technologies GmbH	#AM9516
Chloroform	Carl Roth GmbH	#3313.1
Isopropanol	Carl Roth GmbH	#9781.1
Ethanol	Carl Roth GmbH	#5054.1
**Bulk RNA IAA treatment**		
DMSO	Sigma-Aldrich	#D8418
Ethanol	Carl Roth GmbH	#5054.1
Na_2PO_4	Alfa Alsar	#J60825
DTT	Carl Roth GmbH	#6908.1

© 2022 The Authors

Reagents and Tools table (continued)

Reagent / resource	Reference / source	Identifier /catalog number
**Bulk library preparation**		
PCR primers	Junker *et al* (2014)	
First Strand buffer	Life Technologies GmbH	#18064014
DTT 100 mM	Life Technologies GmbH	#707265
Nuclease free water	Sigma	#W3513
RNaseOut	Life Technologies GmbH	#10777019
Superscript II	Life Technologies GmbH	#18064014
Second-strand buffer	ThermoFisher	#10812014
DNA ligase	New England Biolabs	#M0205L
DNA Pol I	New England Biolabs	#M0209L
RNase H	New England Biolabs	#M0297L
AMPpure XP beads	Beckman Coulter	#19245300
HiScribe T7 kit	New England Biolabs	#E2040S
Exo I	New England Biolabs	#M0293L
rSAP	New England Biolabs	#M0371L
Tris-Acetat pH8.1	Carl Roth GmbH	#71251
Potassium-Acetat	VWR Chemicals	#43763N
Mg-Acetat	Carl Roth GmbH	#HN11.1
RNAclean XP beads	Beckman Coulter	#18816300
NEBNext PCR mix 2×	New England Biolabs	#M0541L
**Methanol fixation**		
Methanol	Th. Geyer	#1437.2511
HBSS	Life Technologies GmbH	#14175095
**Single cell library preparation**		
Single Cell 3' v3.1 kit	10× Genomics	#1000121

Methods and Protocols

Animals

All animal procedures were conducted as approved by the local authorities (LAGeSo, Berlin, Germany).

Zebrafish: Fish were maintained according to standard laboratory conditions. For larval experiments, we set up group crosses of AB wild type fish and the larvae were grown to 48 hpf in egg water at 28°C. The chorions were removed manually at 24 hpf.

Mice: Animals were kept according to the rules for Animal Welfare of the German Society for Laboratory Animal Science under a 12 h/12 h dark–light cycle at 22 ± 2°C and 55 ± 10% humidity with food and water supply *ad libitum*.

Data collection and analysis were not performed blind due to the conditions of the experiments.

Cell culture

On day 1 CD1 feeder cells (derived from CD1 mice, charles river) were seeded at a density of ~ 800 M cells per plate in gelatinized 9 × 6 cm plates with ESCM media (Knockout Dulbecco's Modified Eagle's Medium (DMEM), 4,500 mg/ml glucose, with sodium pyruvate (Gibco, #10829-018), 15% fetal calf serum (FCS; Superior, Biochrom, #S0615), 1× glutamine (100×, Lonza, #BE17-605E), 200 mM, 1× penicillin/streptomycin (100×, Lonza, #DE17-603), 1× nonessential amino acids (Gibco, #11140-35), 1× fresh 10 mM β-mercaptoethanol (Sigma #M-7522; 2-ME), 1× nucleosides (Chemicon #ES-008D)) and incubated at 37°C. On day 2 G4 ES cells (George *et al*, 2007) were seeded on top of the feeder cells (~ 250 M cells per plate) and the medium changed to ESCM + LIF (0.01% LIF Murine Leukemia Inhibitory Factor ESGROTM; 10^7 U/ml, Chemicon, #ESG1107). On day 3 the medium was changed with fresh ESCK + LIF. On day 4 10 mM, 1 mM or 100 µM 4sU were added and the cells incubated for 2 h, 6 h or 24 h.

After the incubation period, the cells were washed twice with PBS (Lonza #BE17-512F) and trypsinized for 7 min at 37C with 1 ml trypsin 0.05% (Gibco #25300-054). Trypsinization was stopped by adding 9 ml ESCM + LIF. Cells were spun down for 5 min at 1,100 rpm at RT and resuspended in the same media (containing 4sU). In order to deplete feeder cells the trypsinized cells were plated again in fresh gelatinized plates and left to attach for 1 h at 37°C. Afterwards the supernatant (containing non-attaching ES cells) was spun down for 5 min at 1,100 rpm and resuspended in 1 ml of TRIzol (Life Technologies GmbH #15596018). The sample was vortexed to facilitate lysis and stored at −20°C.

All cells were tested for mycoplasma prior to culturing.

 © 2022 The Authors

Injections of 4sU into 48 hpf zebrafish larvae

Zebrafish larvae were injected with 5 nl 4sU (100 mM in 10 mM Tris•HCl pH 7.4, (Merck Chemicals GmbH #T4509) with Dextran fluorescein (Thermo Fisher Scientific #7136)) using a glass needle into the duct of Cuvier. Injected larvae were incubated at 28°C for 30 min. Successful injection into the bloodstream was then confirmed by fluorescence microscopy (Leica M165 FC) and larvae showing fluorescence all over the blood vessels were selected. Per experiment we anesthetized 10 selected larvae on ice for 5 min before removing the supernatant and resuspending the larvae in 500 µl TRIzol for homogenization (PerlmühlenHomogenisator Fisher Scientific GmbH).

Labeling of RNA during dissociation

In general, we added 10 mM 4sU to the dissociation buffers and aimed for an incubation time of at least 30 min while continuing to perform the usual steps of each individual dissociation protocol. Detailed protocols were the following.

Dissociation of zebrafish larvae

Ten dechorionated larvae were anesthetized by placing them in 2 ml centrifuge tubes on ice for 5 min. We then washed them twice using ice cold HBSS (Life Technologies GmbH #14175095) and removed the supernatant afterwards. For dissociation, the larvae were either:

- resuspended in 500 µl TryplE Mix (1×TrypleE (Life Technologies GmbH #A1217701) in HBSS, 1 mM EDTA pH 8 (Molekula #900493), 10 mM 4sU for labeled sample) and incubated at 37°C for 30 min (singe cell and bulk).

or

- resuspended in 200 µl psychrophilic protease mix (1× Native Bacillus licheniformis Protease (Creative Enzymes #NATE-0633) in HBSS, 10 mM 4sU) and incubated on ice for 30 min (bulk only). Dissociation was aided by pipetting up and down with a 200 µl tip ~ 25 times every 5 min.

When all visible tissue had disappeared, we inactivated the enzymes by adding 15 µl 10% BSA (Sigma-Aldrich; #A4503) to the suspension and spun down at 1,000 *g* for 5 min (4°C). Finally, the cells were washed once in ice cold HBSS (no 4sU added).

Samples for bulk library preparation (Fig 1) were resuspended in Trizol afterwards.

For the single cell samples (Fig 3), we fixed the first one in Methanol (see below) immediately, while the other two were submitted to FACS sorting with the gates set to include everything apart from very small debris and extremes in size and granularity. The samples were stored on ice for ~ 15 min during FACS setup. Sorting lasted 10 min per sample with no 4sU present in the FACS buffer (HBSS w/1% BSA). 10 mm 4sU was added to the FACS buffer in the cooled receiving tube. Both samples were spun down and washed immediately after their sort before we resuspended the cells in ice cold HBSS (no 4sU added). Sample 2 was fixed in methanol directly afterwards, while sample 3 was left to incubate on ice for another 30 min in order to simulate waiting times when preparing multiple samples. Eventually, sample 3 was also fixed using methanol.

Preparation of adult cardiomyocytes

Single cardiomyocytes were prepared from 10-week-old male wild-type mice maintained on a C57BL/6N background (Jackson Laboratories). Mice were sacrificed by cervical dislocation, the hearts excised and digested via retrograde perfusion with Liberase (Rudolph *et al*, 2019). Perfusion was started with 3 ml/min Perfusion buffer (Stock perfusion buffer (PS00000452), 10 mM 2,3-Butanedionemonoxime (BDM), 5.5 mM Glucose (Sigma-Aldrich #G7528)) for 4 min at 37°C (42°C for sample 5), and then switched to Digestion buffer (Perfusion buffer pH 7.46 (PS00000451), 0.25 mg/ml Liberase blendzyme 1 (Roche #1988417), 0.14 mg/ml trypsin (Gibco/Invitrogen #15090046), 12.5 µM $CaCl_2$) for 8 min. The hearts were transferred from the Langendorff perfusion system to a dish containing 2.5 ml Digestion buffer, where the left ventricle (LV) was cut. After mincing for 2 min, the LV tissue was transferred to a dish with 2.5 ml Stop buffer (Perfusion buffer pH 7.46 (PS00000451), 10% Bovine calf serum (HyClone #SH30073), 12.5 µM $CaCl_2$) and dissociated by pipetting for 4 min. Cardiomyocytes were sedimented by gravity for 10 min. Then, the supernatant was transferred to a new tube and centrifuged for 1 min at 180 *g* to pellet the remaining cardiomyocytes. After discarding the supernatant, the pellet was resuspended in 10 ml Stop buffer and combined with the initial cardiomyocyte pellet. After filtering with a 100 µm strainer and cell counting, the suspension was centrifuged again and the resulting cardiomyocyte pellet was resuspended in 1.3 ml TRIzol and stored at −80°C. In those preparations with 4-Thiouridin, 10 mM 4sU was added to the Digestion and Stop buffers from ventricular mincing to last centrifugation steps before resuspension in TRIzol. The total 4sU incubation time was always 30 min at room temperature, and the untreated controls followed identical handling and step durations. Live, rod-shaped cardiomyocyte counts were in the range of 2.25–7.25 × 10^5 per preparation.

Preparation of embryonic cardiomyocytes

Pregnant C57BL/6N (Jackson Laboratories) female mice were sacrificed by cervical dislocation. E17.5 embryos were removed, rinsed in PBS and transferred to a 35-mm dish with cold PBS. Embryonic hearts were microdissected away from the embryo and placed in another dish with PBS and 10 mM 4sU. The hearts were finely minced with a scalpel and transferred into a tube with 1.5 ml pre-warmed 0.25% trypsin–EDTA (Thermo Fisher Scientific #25200056) and 10 mM 4sU. The hearts were digested for 15 min at 37°C with several rounds of vigorous pipetting and then centrifuged at 400 *g* for 3 min. The cell pellet was washed twice in 20% FCS in PBS solution, centrifuged and resuspended in 1 ml PBS. The remaining cell suspension was incubated for 30 min in the dark with 1 µl reconstituted fluorescent reactive dye (Thermo Fisher Scientific #L34955) to determine cell viability. After another wash in PBS, cells were resuspended in 0.5 ml 5% FCS in PBS solution. The cell suspensions were then incubated with 1 µg/ml Alexa Fluor 647 Rat Anti-Mouse VCAM-1 (BD Biosciences #561612) for 15 min in the dark. The cells were centrifuged at 450 *g* for 5 min, resuspended in PBS and filtered using a 70 µm strainer into a polypropylene tube. Live, VCAM-1^+ cardiomyocytes were sorted using a BD FACSAria II (BD Biosciences), transferred to 1.5 ml tubes and centrifuged at 450 *g* for 5 min. The cell pellets were resuspended in 1 ml TRIzol and stored at −80°C. All steps after embryo removal were done at 4°C except

© 2022 The Authors

for tissue digestion with trypsin (37°C). The total 4sU incubation time was always for 30–40 min.

Microglia and hippocampus cell isolation

All mice used in this study were based on a C57BL/6 background and aged 10–14 weeks. Animals were perfused with PBS before the brain extraction. Cells were isolated using the Adult Brain Dissociation Kit (ABDK, Miltenyi Biotec #130-107-677). In brief, mice were anesthetized (Narcorene, 100 mg/kg, i.p., Merial GmbH, Germany) and perfused transcardially with 10 ml of 0.9% saline solution containing 1 U/ml of heparin (Heparin-Natrium-250,000-ratiopharm, Ratiopharm, Ulm, Germany) followed by 50 ml of 0.1 M PBS (pH 7.4). Next, the brains were gently extracted from the skull (Papouin & Haydon, 2018) and immediately placed in ice cold HBSS. For the hippocampus isolation, brains where put on an ice cold metal platform and the hippocampus was dissected out of the whole brain as described by Spijker, 2011. Hippocampi were then placed in the ABDK enzyme mix and dissociated for 30 min at 37°C according to the manufacturer's instructions in the presence of 10 mM 4sU. Afterwards the cell suspension underwent myelin removal with 35% SIP (Solution Isotonic Percoll) for 25 min at 4°C. Live cells were sorted next with all steps being performed on ice and/or with pre-chilled buffers and equipment at 4°C. Cells were stained with BD Pharmingen™ Calcein AM dye for live/dead cell staining (BD Pharmingen #564061) at 10 μM final concentration for 30-40 min on ice. Afterwards the cell suspension was spun down for 5 min at 300 *g*, before being resuspended in 500 μl of FACS buffer. Next, cells were sorted using an ARIA II sorter with 70 μl nozzle at the speed of 10 k cells/s.

Bulk library preparation

RNA extraction

Bulk RNA was extracted using the Trizol-chloroform-isopropanol method as described in Junker *et al*, 2014. In brief, we added 0.5 μl GlycoBlue (Life Technologies GmbH #AM9516) to 500 μl of sample in Trizol, mixed well and incubated the tubes at RT for 15 min before adding 100 μl of Chloroform (Carl Roth, #3313.1). After shaking vigorously, the samples were incubated at RT for 5 min and then centrifuged for 15 min at 12,000 *g* in a centrifuge cooled to 4°C to aid phase separation. We carefully transferred 250 μl of the aqueous phase to a new tube, added 250 μl of isopropanol (Carl Roth, #9781.1), shook well and incubated the samples over night at −20°C.

The next morning the samples were centrifuged for 10 min at 12,000 *g* (4°C) and the resulting pellets washed in 500 μl 70% ethanol. Finally, we resuspended the RNA in RNase free water to continue with IAA treatment.

IAA treatment

Based on Herzog *et al*, 2017 we performed an IAA derivatization treatment at 50°C for 15 min in 50% DMSO (Sigma-Aldrich #D8418) / 10% ethanol / 45% water with 10 mM IAA (Merck Chemicals GmbH #11149) and 50 mM Na_2PO_4 (Alfa Alsar #J60825) present. After IAA treatment, we stopped the reaction by adding DTT (Carl Roth, #6908.1; 40 mM final) and extracted the RNA using another round of RNA extraction in Trizol-chloroform-isopropanol as described above.

Library preparation

Bulk libraries were prepared using a slightly modified version of the protocol published in Junker *et al* (2014). Briefly, we first generated cDNA from up to 50 ng of input material using barcoded primers. Primers (5.4 ng/μl with dNTPs 1 mM each) were annealed for 5 min at 65°C before adding 2 μl RT mix to 2 μl of sample/primer (0.8 μl First Strand buffer; Life Technologies GmbH #18064014), 0.5 μl DTT 100 mM (Life Technologies GmbH #707265), 0.4 μl nuclease free water (Sigma #W3513), 0.2 μl RNaseOut (Life Technologies GmbH #10777019), 0.2 μl superscript II (Life Technologies GmbH #18064014). The samples were incubated at 42°C for 1 h in a thermal cycler with the lid set to 50°C. Afterwards the enzyme was heat inactivated for 10 min at 70°C.

Second strand synthesis was carried out at 16°C for 1 h after adding 9 μl of second strand mix (5.72 μl Nuclease-free water, 2.5 μl Second-strand buffer (ThermoFisher #10812014), 0.25 μl dNTPs (10 mM total), 0.09 μl DNA ligase (New England Biolabs #M0205L), 0.35 μl *E. coli* DNA Pol I (New England Biolabs #M0209L), 0.09 μl RNase H (New England Biolabs #M0297L)) to the sample. If multiple samples were processed they could be pooled at this stage. We then cleaned the cDNA by adding 0.2 μl AMPpure XP beads (Beckman Coulter #19245300) and 1 μl of bead binding buffer per 1 μl of sample. The resulting cDNA was amplified by *in vitro* transcription using the HiScribe T7 kit (New England Biolabs #E2040S) for 16 h at 37°C.

Residual primers were removed by adding 3 μl Exo I (New England Biolabs #M0293L) and 3 μl rSAP (New England Biolabs #M0371L) to 16 μl of aRNA. Afterwards we fragmented the aRNA by adding 5.5 μl of fragmentation buffer (200 mM Tris-Acetat pH8.1 (Carl Roth GmbH #71251), 500 mM Potassium-Acetat (VWR Chemicals #43763N), 150 mM Mg-Acetat (Carl Roth GmbH #HN11.1)) to the resulting 22 μl of sample and heating to 94°C for 3–6 min. The fragmented aRNA was then cleaned using 1.8× RNAclean XP beads (Beckman Coulter #18816300) and measured using a Qubit 4 Fluorometer (Life Technologies GmbH # q33226) system as well as a TapeStation 4200 (Agilent) high sensitivity RNA device.

A maximum of 250 ng of aRNA was carried over to second RT. 0.5 μl of dNTPs (10 mM total) and 0.5 μl of second RT primer were added to 5 μl of RNA and annealed at 65°C for 5 min. Afterwards second RT mix (5 μl RNA in nuclease free water, 2 μl Second Strand buffer, 1 μl DTT 100 mM, 0.5 μl RNaseOut, 0.5 μl Superscript II) was added and incubated in a thermal cycler for 10 min at 25°C, and then for 1 h at 42°C. Enzymes were inactivated for 10 min at 70°C.

Eventually, we added library PCR mix (10 μl nuclease free water, 25 μl NEBNext PCR mix 2× (New England Biolabs #M0541L), 2.5 μl RP1 primer (10 μM), 2.5 μl RPI index primer (10 μM)) and amplified using the following program:

30 s at 98°C.
12–15 cycles of:
10 s at 98°C.
30 s at 62°C.
30 s at 72°C.
10 min at 72°C.
hold at 10°C.

Libraries were cleaned using AMPpure XP DNA beads at 0.9× followed by 1.0×. Libraries were quantified using the Qubit system as

© 2022 The Authors

well as a TapeStation D5000 HS device and sequenced on a NextSeq 500 system (Illumina).

Single cell methods

MeOH fixation and IAA treatment

As described in Holler *et al*, 2021, cells were spun down and resuspended in HBSS, before cold methanol (Th. Geyer #1437.2511) was added drop-wise while gently agitating on a vortexer. Afterwards the cells were fixed at −20°C for at least 30 min. For IAA treatment, the samples were brought back to RT and IAA dissolved in 20% HBSS / 80% MeOH was added to a final concentration of 10 mM IAA. The samples were then set to rotate at 1 rpm at RT for 13 h (mouse hippocampus) or 16 h (zebrafish larvae) while taped lengthwise to a rotator to avoid splashing and undue strain on the cells.

Rehydration and library preparation of zebrafish larvae cells

Methanol fixed cells were rehydrated using DPBS as described in Holler *et al* (2021).

Briefly, we inactivated the IAA, by spinning down at 1,000 *g* for 5 min and resuspending the cells in quenching buffer (DBPS, Gibco #14190144, 0.1% BSA, 1 U/µl RNaseOUT, Life Technologies #10777019, 100 mM DTT, Carl Roth #6908.1). The samples were incubated at room temperature for 5 min. After spinning down again, we resuspended them in DPBS containing 0.01% BSA, 0.5 U/µl RNaseOUT and 1 mM DTT. Afterwards the cells were then passed through a 35 µm strainer, counted, and immediately loaded onto a 10× Chromium Single Cell 3′ v3.1 kit (10× Genomics #1000121).

Libraries were prepared according to the manufacturer's instructions and sequenced on an Illumina NovaSeq system to a depth of ~ 400 Mio reads per library.

Rehydration and library preparation of mouse hippocampus cells

Cells were rehydrated using 3× SSC buffer based on the recommendations by 10× Genomics (https://support.10xgenomics.com/permalink/4sImBCtiKcsk6SkwQQuCGM). We modified the procedure to accommodate for SLAM-seq as follows:

First the cells were spun down at 1,000 *g* and 4°C for 10 min, the supernatant was removed and quenching buffer added (3× SSC (Carl Roth GmbH #1054.1), 0.1% BSA, 1 U/µl SuperRNase IN (Life Technologies GmbH #AM2694), 100 mM DTT). We incubated the sample at RT for 5 min on order to quench the remaining IAA. Afterwards the cells were spun down again and resuspended in a small amount of resuspension buffer (1.5× SSC, 0.5 U/µl SuperRNase IN, 0.01% BSA) and loaded onto the chromium controller using a Chromium Single Cell 3′ v3.1 kit (10× Genomics #1000121). Importantly, the volume of SSC buffer introduced into reverse transcription needs to be as low as possible to avoid inhibiting the reaction.

After successful encapsulation, we proceeded with library preparation according to the manufacturer's instructions. The libraries were sequenced on a NextSeq 500 with about 50–60 Mio reads per library.

Computational methods

Mapping and substitution identification

Reads were demultiplexed using bcl2fastq v2.19. Bulk data was mapped using STAR v2.7.3a to either dr11.95 (zebrafish) or mm38 (Ensembl 100, mouse) including reference information on substitutions (MD tag). Single cell data was mapped using cellranger v6.1.1 to mm38 (Ensembl 98, mouse) or GRCz10 (zebrafish). MD tags were added using samtools v1.14 afterwards.

In the next step, we aggregated information on substitution type, location and sequencing quality as an additional tag (MT tag) in order to save computation time in downstream steps. For quality filtering, we regarded a sequencing quality of 20 as sufficient. Bulk data was annotated using Rsubread (Liao *et al*, 2019).

SNP removal and labeling criteria for bulk data

SNPs were removed by blacklisting all genomic positions where more than 25% of all reads deviated from the reference base. Positions with a total coverage below 10 were blacklisted as well. Labelling rates per gene were then calculated by determining the fraction of non-blacklisted T-positions that showed T to C mutations of sufficient quality. Genes with labeling rates over 5 standard deviations away from the mean of all genes are highlighted in blue and were used for GO term analysis. PCA was carried out on T to C rates of all genes found in both adult and all three prenatal samples which had a T to C rate at least one standard deviation over the mean in at least one sample (in order to reduce noise).

Clustering of single cell data

Single cell data was clustered using Seurat (Hao *et al*, 2021) v4.0.4. For the single cell zebrafish data shown in Fig 3, the datasets were merged after normalizing, scaling and clustering each one individually. Cell types were determined based on marker gene expression for each data set individually before transferring the labels to the merged data set. Minor cell types only present in one or two data sets were removed for clarity (with the exception of muscle cells, which were the only cell type exclusively present in the non-FACSed dataset).

Removal of labeled UMIs in single cell data

After initial clustering, labeled UMIs were removed based on T to C mutations of a sequencing quality of at least 20. Afterwards count matrices were re-generated using custom code and again clustered using the same settings in Seurat.

Labeling rates in removed UMIs were calculated by determining the fraction of T-positions that showed T to C mutations of sufficient quality.

GO-term analysis (for bulk and single cell data)

GO-term analysis was performed on genes with labeling rates over 5 standard deviations away from the mean of all genes (bulk data) or all genes that have more than 3 reads with 2 or more labeling events (mouse single cell data) using the generic gene ontology term finder provided by Princeton University (https://go.princeton.edu/cgi-bin/GOTermFinder).

Step-by-step protocol

Materials

Reagents and supplies for the tissue specific dissociation protocol.

4sU (Merck Chemicals GmbH).

Iodoacetamide (Merck Chemicals GmbH).

Additionally for bulk samples:

RNA extraction supplies (Trizol-chloroform-isopropanol based extraction tested).

DMSO (Sigma-Aldrich).

Ethanol.

Na_2PO_4 (Alfa Alsar).

© 2022 The Authors

Additionally for single cell samples:

HBSS (Life Technologies GmbH).
Methanol (Th. Geyer).
3× SSC (Carl Roth GmbH) *or* DPBS (Gibco).
BSA (Sigma-Aldrich).
RNAse Inhibitor (RNaseOUT, Life Technologies *or* SuperRNase IN, Life Technologies GmbH).
DTT (Carl Roth GmbH).

Dissociation

Dissociate samples as usual but supplement buffers with 10 mM 4sU for at least 30 min of the dissociation procedure to allow for 4sU uptake. Limit exposure to light as much as possible as 4sU is light sensitive.

The exact concentration of 4sU may need to be titrated for different tissues to reach a balance between deeper penetration and potential toxicity.

For bulk samples:

After dissociation (and cell sorting, if desired), extract RNA using your method of choice, again limit exposure to light.
Resuspend extracted RNA in RNAse free water (with no reducing agent added!) and start IAA treatment as soon as possible.

Based on Herzog *et al* set up the following reaction:

15 µl RNA in H2O.
25 µl DMSO.
5 µl 100 mM IAA in EtOH.
5 µl 500 mM Na_2PO_4.
and heat to 50°C for 15 min in a thermocycler. Put on ice and stop the reaction using 1 µl of 1 M DTT.

Proceed with another round of RNA extraction.

For single cell samples:

Resuspend cells in HBSS and add cold Methanol dropwise to 80% final volume while vortexing slowly.

Set samples to −20°C for at least 30 min to fix.

Add IAA in 20% HBSS/80% MeOH to 10 mM final concentration and set samples to gently agitate at room temperature over night. Avoid vigorous shaking/splashing as it might damage the cells.

After 13 to 16 h rehydrate the fixed cells either using DPBS (as described in Holler *et al*, 2021) or using 3× SSC (based on 10× Genomics recommendations; https://support.10xgenomics.com/permalink/4sImBCtiKcsk6SkwQQuCGM).

For both versions, there is an IAA quenching step (5 min at RT in DTT high buffer) and a subsequent resuspension step (DTT low buffer to pass into the single cell application of choice).

Using DPBS has the advantage of being relatively low in salt and therefore not interfering with reverse transcription whereas a cell suspension prepared in 3×SSC needs to be heavily diluted right before reverse transcription in order to mitigate reverse transcription inhibition. 3×SSC might be more beneficial to cell integrity in some samples, so test accordingly.

For the DPBS protocol:

Spin down at max. 1,000 *g* for 5–10 min at 4°C (adjust speed and duration in case of cell damage), remove the supernatant and resuspend in quenching buffer (DBPS, Gibco; 0.1% BSA, Sigma-Aldrich; 1 U/µl RNaseOUT, Life Technologies; 100 mM DTT, Carl Roth).
Incubate at room temperature for 5 min.
Spin down again (same parameters as before) and resuspend sample in pre-cooled resuspension buffer (DPBS, 0.01% BSA, 0.5 U/µl RnaseOUT, 1 mM DTT – required by RnaseOUT).
Keep sample on ice.
Pass through a strainer if needed for downstream application, count cells and proceed to single cell library preparation as fast as possible to avoid cell agglomerates forming.

For the 3×SSC protocol:

Spin samples down at 1,000 *g* and 4°C for 5–10 min, remove the supernatant and resuspend in quenching buffer (3× SSC, Carl Roth GmbH; 0.1% BSA; 1 U/µl SuperRNase IN, Life Technologies GmbH; 100 mM DTT).
Incubate at room temperature for 5 min.
Spin down again (same parameters as before) and resuspend sample in pre-cooled resuspension buffer (1.5× SSC, 0.5 U/µl SuperRNase IN, 0.01% BSA). SSC concentration can be adjusted up to 3× SSC if cell numbers allow higher dilution factors downstream. The amount of SSC introduced into reverse transcription needs to be as low as possible in avoid inhibiting the reaction.
Pass through a strainer if needed for downstream application, count cells and proceed to single cell library preparation as fast as possible to avoid cell agglomerates forming.

Analysis

Custom software is available at github as described in the data availability section, or alternatively use other published SLAM-seq software.

Data availability

The datasets and computer code produced in this study are available in the following databases:

- RNA-seq data: Gene Expression Omnibus GSE202949 (https://www.ncbi.nlm.nih.gov/geo/query/acc.cgi?acc=GSE202949).
- Custom code: GitHub (https://github.com/anikaneuschulz/MTglob_pipeline).

Expanded View for this article is available online.

Acknowledgements
We acknowledge support by MDC/BIMSB core facilities (mouse and zebrafish facilities, genomics, bioinformatics, flow cytometry), we thank I. Gomez-Argente for help with cell culture experiments, J. Richter, R. Schäfer, J. Mintcheva and N. Mitic for support with zebrafish experiments, and A. Eisenmann, M. Radke and H.-P. Rahn for expert technical assistance. Work in JPJ's laboratory was funded by a Multiscale Omics grant from the Berlin Institute of Health (BIH), by Helmholtz AI grant SC-SLAM-ATAC (ZT-I-PF-5-54), and by the DZHK (Berlin partner site project 81Z0100112). MG's laboratory is supported

© 2022 The Authors

by a grant from the Leducq foundation and the DFG. AN was supported by a PhD fellowship from *Studienstiftung des deutschen Volkes*. Artwork in Figs 1, 4 and EV1 was created using BioRender.com. Open access funding enabled and organized by Projekt DEAL.

Author contributions

Jan Philipp Junker: Conceptualization; funding acquisition; writing – original draft; project administration; writing – review and editing. **Anika Neuschulz:** Conceptualization; data curation; software; formal analysis; validation; investigation; visualization; methodology; writing – original draft; writing – review and editing. **Olga Bakina:** Investigation; writing – original draft. **Victor Badillo-Lisakowski:** Investigation; writing – original draft. **Pedro Olivares-Chauvet:** Formal analysis; methodology. **Thomas Conrad:** Investigation; methodology. **Michael Gotthardt:** Supervision. **Helmut Kettenmann:** Supervision.

Disclosure and competing interests statement

The authors declare that they have no conflict of interest.

References

Bensaude O (2011) Inhibiting eukaryotic transcription. Which compound to choose? How to evaluate its activity? Which compound to choose? How to evaluate its activity? *Transcription* 2: 103 – 108

van den Brink SC, Sage F, Vértesy Á, Spanjaard B, Peterson-Maduro J, Baron CS, Robin C, van Oudenaarden A (2017) Single-cell sequencing reveals dissociation-induced gene expression in tissue subpopulations. *Nat Methods* 14: 935 – 936

Cao J, Zhou W, Steemers F, Trapnell C, Shendure J (2020) Sci-fate characterizes the dynamics of gene expression in single cells. *Nat Biotechnol* 38: 980 – 988

Cassé C, Giannoni F, Nguyen VT, Dubois M-F, Bensaude O (1999) The transcriptional inhibitors, actinomycin D and α-Amanitin, activate the HIV-1 promoter and favor phosphorylation of the RNA polymerase II C-terminal domain. *J Biol Chem* 274: 16097 – 16106

Denisenko E, Guo BB, Jones M, Hou R, de Kock L, Lassmann T, Poppe D, Clément O, Simmons RK, Lister R *et al* (2020) Systematic assessment of tissue dissociation and storage biases in single-cell and single-nucleus RNA-seq workflows. *Genome Biol* 21: 130

Erhard F, Baptista MAP, Krammer T, Hennig T, Lange M, Arampatzi P, Jürges CS, Theis FJ, Saliba A-E, Dölken L (2019) scSLAM-seq reveals core features of transcription dynamics in single cells. *Nature* 571: 1 – 21

George SHL, Gertsenstein M, Vintersten K, Korets-Smith E, Murphy J, Stevens ME, Haigh JJ, Nagy A (2007) Developmental and adult phenotyping directly from mutant embryonic stem cells. *Proc Natl Acad Sci U S A* 104: 4455 – 4460

Hammond TR, Dufort C, Dissing-Olesen L, Giera S, Young A, Wysoker A, Walker AJ, Gergits F, Segel M, Nemesh J *et al* (2019) Single-cell RNA sequencing of microglia throughout the mouse lifespan and in the injured brain reveals complex cell-state changes. *Immunity* 50: 253 – 271

Hao Y, Hao S, Andersen-Nissen E, Mauck WM, Zheng S, Butler A, Lee MJ, Wilk AJ, Darby C, Zager M *et al* (2021) Integrated analysis of multimodal single-cell data. *Cell* 184: 3573 – 3587

Hendriks G-J, Jung LA, Larsson AJM, Lidschreiber M, Forsman OA, Lidschreiber K, Cramer P, Sandberg R (2019) NASC-seq monitors RNA synthesis in single cells. *Nat Commun* 10: 3138

Herzog VA, Reichholf B, Neumann T, Rescheneder P, Bhat P, Burkard TR, Wlotzka W, von Haeseler A, Zuber J, Ameres SL (2017) Thiol-linked alkylation of RNA to assess expression dynamics. *Nat Methods* 14: 1198 – 1204

Holler K, Neuschulz A, Drewe-Boß P, Mintcheva J, Spanjaard B, Arsiè R, Ohler U, Landthaler M, Junker JP (2021) Spatio-temporal mRNA tracking in the early zebrafish embryo. *Nat Commun* 12: 3358

Hrvatin S, Hochbaum DR, Nagy MA, Cicconet M, Robertson K, Cheadle L, Zilionis R, Ratner A, Borges-Monroy R, Klein AM *et al* (2018) Single-cell analysis of experience-dependent transcriptomic states in the mouse visual cortex. *Nat Neurosci* 21: 120 – 129

Junker JP, Noël ES, Guryev V, Peterson KA, Shah G, Huisken J, McMahon AP, Berezikov E, Bakkers J, van Oudenaarden A (2014) Genome-wide RNA tomography in the zebrafish embryo. *Cell* 159: 662 – 675

Keren-Shaul H, Spinrad A, Weiner A, Matcovitch-Natan O, Dvir-Szternfeld R, Ulland TK, David E, Baruch K, Lara-Astaiso D, Toth B *et al* (2017) A unique microglia type associated with restricting development of Alzheimer's disease. *Cell* 169: 1276 – 1290

Lacar B, Linker SB, Jaeger BN, Krishnaswami SR, Barron JJ, Kelder MJE, Parylak SL, Paquola ACM, Venepally P, Novotny M *et al* (2016) Nuclear RNA-seq of single neurons reveals molecular signatures of activation. *Nat Commun* 7: 11022

Liao Y, Smyth GK, Shi W (2019) The R package Rsubread is easier, faster, cheaper and better for alignment and quantification of RNA sequencing reads. *Nucleic Acids Res* 47: e47

Marsh SE, Walker AJ, Kamath T, Dissing-Olesen L, Hammond TR, de Soysa TY, Young AMH, Murphy S, Abdulraouf A, Nadaf N *et al* (2022) Dissection of artifactual and confounding glial signatures by single-cell sequencing of mouse and human brain. *Nat Neurosci* 25: 306 – 316

Mattei D, Ivanov A, van Oostrum M, Pantelyushin S, Richetto J, Mueller F, Beffinger M, Schellhammer L, vom Berg J, Wollscheid B *et al* (2020) Enzymatic dissociation induces transcriptional and proteotype bias in brain cell populations. *Int J Mol Sci* 21: 7944

Ochocka N, Segit P, Walentynowicz KA, Wojnicki K, Cyranowski S, Swatler J, Mieczkowski J, Kaminska B (2021) Single-cell RNA sequencing reveals functional heterogeneity of glioma-associated brain macrophages. *Nat Commun* 12: 1151

Olah M, Menon V, Habib N, Taga MF, Ma Y, Yung CJ, Cimpean M, Khairallah A, Coronas-Samano G, Sankowski R *et al* (2020) Single cell RNA sequencing of human microglia uncovers a subset associated with Alzheimer's disease. *Nat Commun* 11: 6129

Papouin T, Haydon PG (2018) Obtaining acute brain slices. *Bio Protoc* 8: e2699

Qiu Q, Hu P, Qiu X, Govek KW, Cámara PG, Wu H (2020) Massively parallel and time-resolved RNA sequencing in single cells with scNT-seq. *Nat Methods* 17: 991 – 1001

Rudolph F, Hüttemeister J, da Silva LK, Jüttner R, Yu L, Bergmann N, Friedrich D, Preibisch S, Wagner E, Lehnart SE *et al* (2019) Resolving titin's lifecycle and the spatial organization of protein turnover in mouse cardiomyocytes. *Proc Natl Acad Sci U S A* 116: 25126 – 25136

Schwabe D, Formichetti S, Junker JP, Falcke M, Rajewsky N (2020) The transcriptome dynamics of single cells during the cell cycle. *Mol Syst Biol* 16: e9946

Spijker S (2011) Dissection of rodent brain regions. In *Neuroproteomics. Neuromethods*, Li K (ed), Vol. 57. Totowa, NJ: Humana Press

Wu YE, Pan L, Zuo Y, Li X, Hong W (2017) Detecting activated cell populations using single-cell RNA-Seq. *Neuron* 96: 313 – 329

Zeisel A, Hochgerner H, Lönnerberg P, Johnsson A, Memic F, van der Zwan J, Häring M, Braun E, Borm LE, La Manno G *et al* (2018) Molecular architecture of the mouse nervous system. *Cell* 174: 999 – 1014

License: This is an open access article under the terms of the Creative Commons Attribution License, which permits use, distribution and reproduction in any medium, provided the original work is properly cited.

© 2022 The Authors

Expanded View Figures

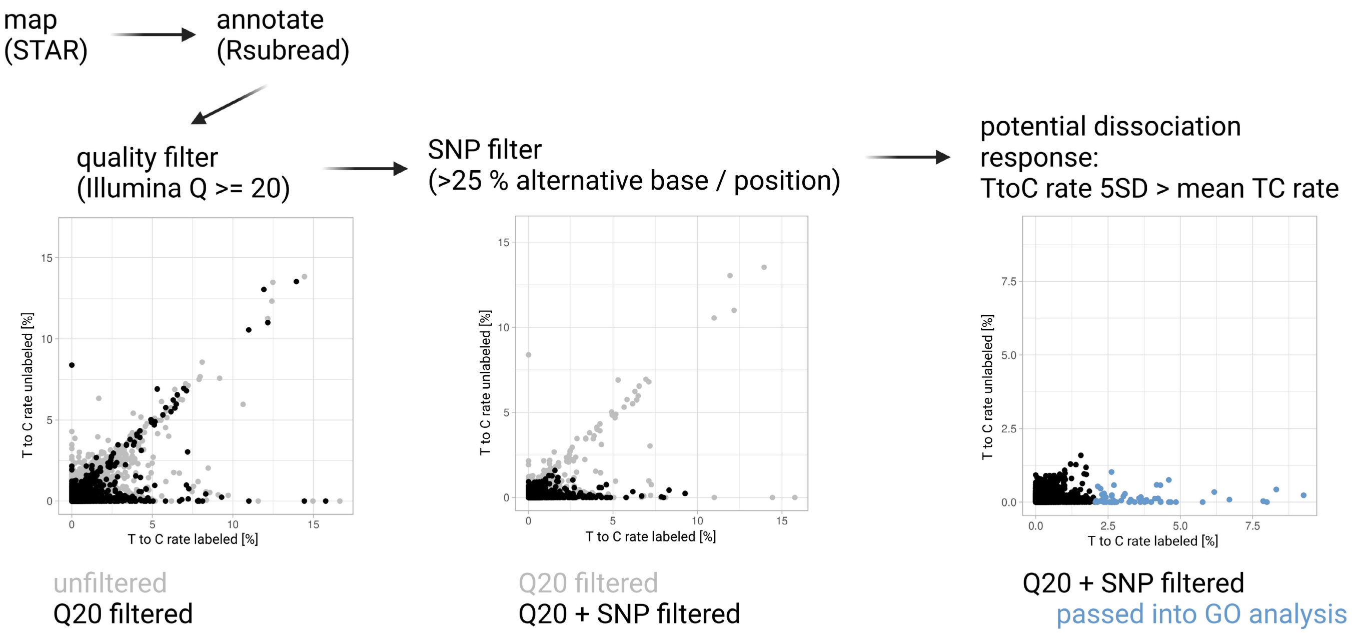

Figure EV1. Computational workflow for bulk data.

Reads are mapped using STAR, annotated using Rsubread and passed through quality and SNP filtering steps before selecting genes for GO analysis based on a T-to-C conversion rate at least 5 standard deviations larger than the mean.

 © 2022 The Authors

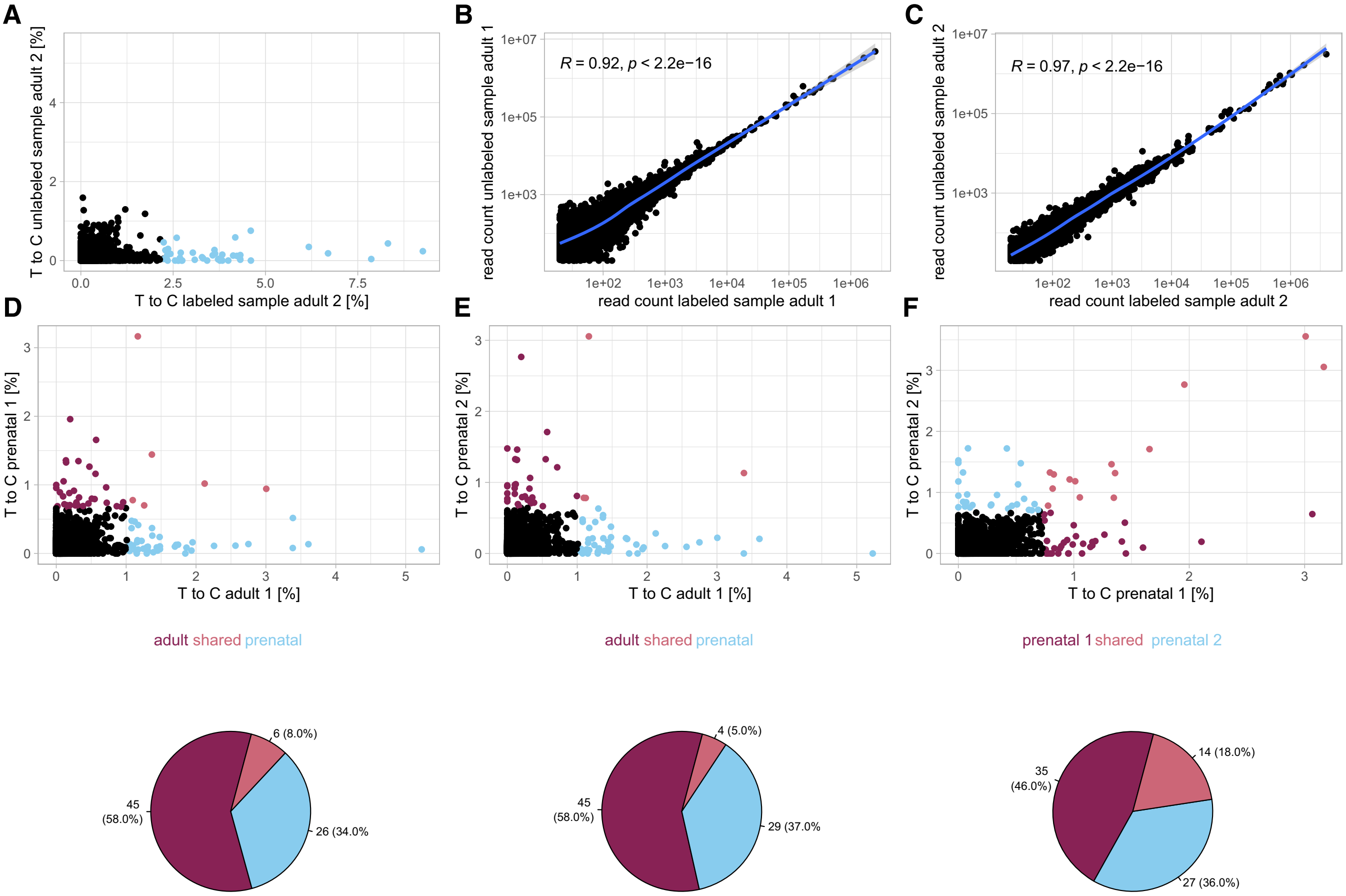

Figure EV2. Biological replicates for cardiomyocyte dissociation experiments.

A Adult mouse cardiomyocytes, replicate 2, T-to-C rates per gene in labeled (4sU added) and unlabeled (no 4sU added) sample. Blue (~ 1% of genes): T-to-C rate ≥ 5 standard deviations.

B, C Read count correlation between labeled and unlabeled adult mouse cardiomyocyte samples. Blue lines show sliding window average (smoothed conditional mean) and 95% confidence interval. *R* values calculated through Pearson correlation.

D, E T-to-C rates per gene in prenatal vs. adult cardiomyocytes. Genes with T-to-C rate ≥ 5 standard deviations in one or both samples are highlighted.

F T-to-C rates per gene in prenatal replicate 1 vs. prenatal replicate 2. Genes with T-to-C rate ≥ 5 standard deviations in one or both samples are highlighted.

© 2023 The Authors

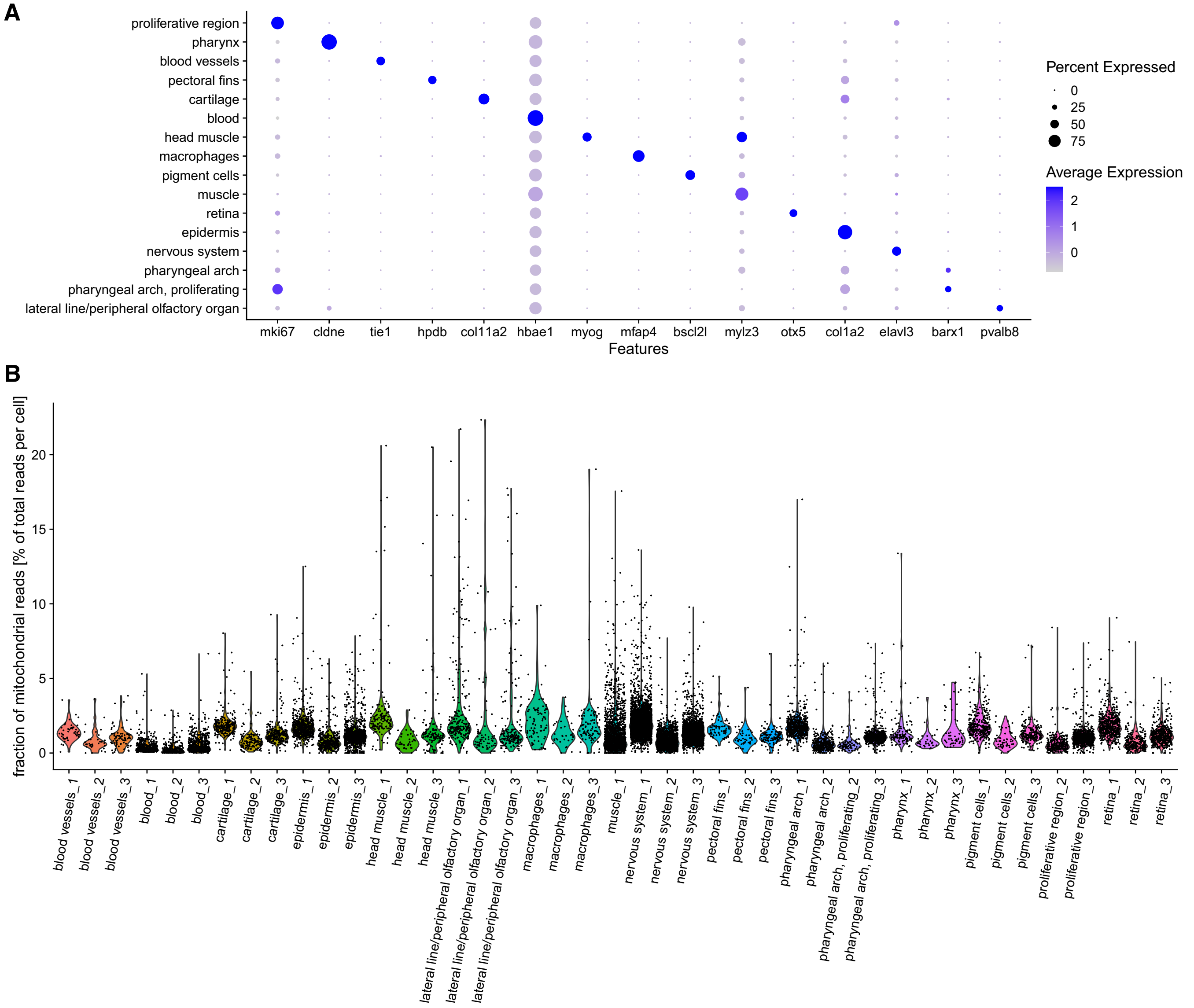

Figure EV3. Characterization of single cell zebrafish data.

A Marker gene expression in 48 hpf zebrafish cells, data from all three samples is merged.

B Fraction of mitochondrial reads per cell, cell types are separated by sample of origin.

 © 2022 The Authors

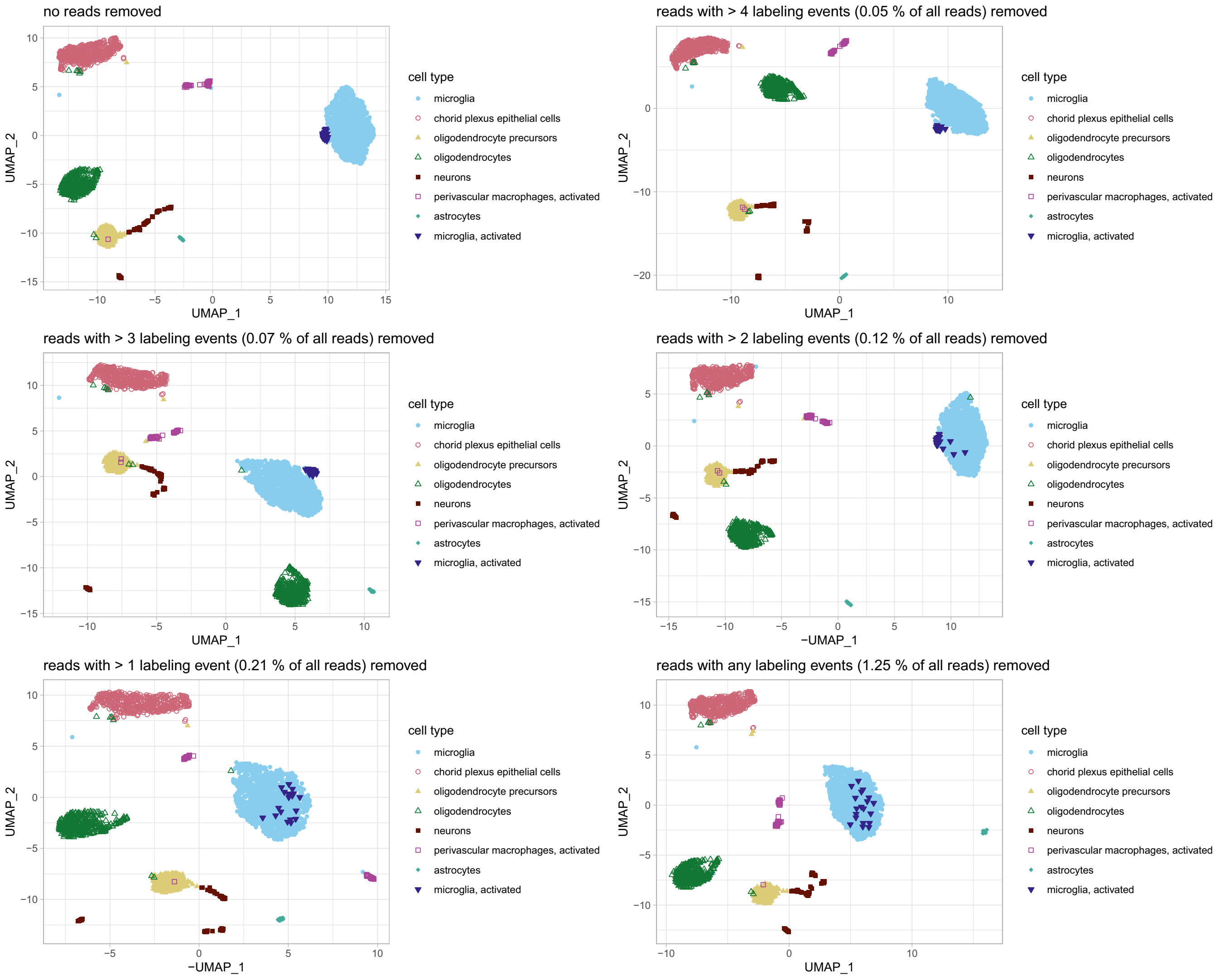

Figure EV4. Dependence of clustering results on labeled transcripts.

Gradual removal of labeled UMIs leads to the activated microglia cluster merging with the non-activated microglia.
