# Supplementary material for: A single‐cell RNA labeling strategy for measuring stress response upon tissue dissociation
Source: Mol Syst Biol. 2022 Dec 27;19(2):e11147. doi: 10.15252/msb.202211147 (PMC9912023; doi:10.15252/msb.202211147)
Supplement: Supplementary file 8 [file MSB-19-e11147-s008.pdf]

molecular systems biology

A single-cell RNA labeling strategy for measuring stress response upon tissue dissociation

Jan Philipp Junker, Anika Neuschulz, Olga Bakina, Victor Badillo-Lisakowski, Pedro Olivares, Thomas Conrad, Michael Gotthardt, and Helmut Kettenmann
DOI: 10.15252/msb.202211147

Corresponding author(s): Jan Philipp Junker (janphilipp.junker@mdc-berlin.de)

Review Timeline:

Submission Date:	29th May 22
Editorial Decision:	27th Jun 22
Revision Received:	9th Nov 22
Editorial Decision:	8th Dec 22
Revision Received:	12th Dec 22
Accepted:	13th Dec 22

Editor: Maria Polychronidou

Transaction Report:

(Note: With the exception of the correction of typographical or spelling errors that could be a source of ambiguity, letters and reports are not edited. Depending on transfer agreements, referee reports obtained elsewhere may or may not be included in this compilation. Referee reports are anonymous unless the Referee chooses to sign their reports.)

Thank you again for submitting your work to Molecular Systems Biology. We have now heard back from the two reviewers who agreed to evaluate your study. As you will see below, the reviewers think that the study is likely to be relevant for those performing scRNA-seq experiments. However, they raise a series of concerns, which we would ask you to address in a revision.

Without repeating all the comments listed below, a couple of important points are the following:

- Reviewer #2 points out that it is required to provide a concrete signature/procedure that can be applied across datasets and allows generalizing the proposed approach and correcting for dissociation effects across experiments.

- As reviewer #1 mentions, including a step-by-step protocol would significantly enhance the impact of the study (see also editorial comments below).

All issues raised by the referees would need to be satisfactorily addressed. Please let me know in case you would like to discuss in further detail any of the issues raised, I would be happy to schedule a call.

On a more editorial level, we would ask you to address the following points:

Reviewer #1:

Analyzing tissue composition at the single-cell resolution is of paramount importance. While spatial transcriptomics is growing up, the sensitivity in capturing RNA molecules, the spatial resolution and the extent of area that can be analysed remain limited. Instead, tissue dissociation followed by single-cell RNA-seq remains the method of choice and the sensitivity of current single-cell RNA-seq protocols is very good as thousands of transcripts across thousands of cells can be captured. However many publications have so far demonstrated that tissue dissociation leads to the activation of stress genes (see for example PMID: 32487174) but yet no generic method exists to precisely measure dissociation artifacts.

The study proposed by Neuschulz et al. proposes to fill this gap by using RNA metabolic labeling to specifically label the genes induced during tissue dissociation. Briefly, tissue dissociation is done along with 4sU (4-thiouridine) RNA metabolic labeling to mark specifically the genes that are transcribed during the procedure. The authors have demonstrated that the procedure is working on zebrafish larvae (Fig 1c,d), mouse cardiomyocytes and mouse hippocampus. The approach is fully compatible with single-cell SLAM-seq allowing to track the dissociation effects at the single-cell level (Fig.2). The authors could show (Fig 1f) that the 'same' dissociation protocol of applied to biological replicates leads to different responses highlighting again the need to implement a robust method to estimate tissue dissociation artifacts. The manuscript is very clear and the experiments are very well conducted. The manuscript will reach a very large audience.

I have a few points that I would like the authors to address mainly in the discussion and suppl. material:

- For the next step, it is very important to make this study a generic study to be implemented in any lab including a lab with poor results that never worked with SLAM-seq. If the author can add a step-by-step protocol it would be very important.

- How methanol fixation affects the procedure? Does it lead to RNA leakage?

- The concentration of 4sU used is very high (10 mM) (Fig 1b). We usually use concentrations in the range of hundreds of uM. Can the author explain this concentration?

- How does the 'dissociation-specific genes' found here are comparable to genes found in other reports?

- Does cold dissociation lead to dissociation artifacts? If the authors can add an experiment it would be great.

Minor comments:
- It would be informative if the authors can add systematically the number of genes that are tissue dissociation specific. Example: line 71 instead of writing 'a smaller number of genes', it would be preferable to give the numbers.
- Fig 1g: it would be important to add in the legend what genes were used to calculate the PCA.

Reviewer #2:

Review: MSB-2022-11147
A single-cell RNA labeling strategy for measuring stress response upon tissue dissociation

Summary

The authors provide a general strategy for isolating the effects of tissue dissociation on transcript abundances. Taking advantage of metabolic labeling techniques that identify newly synthesized transcripts, the authors focus on quantifying a potential source of technical variation in single-cell RNA-seq datasets. The results in this work ought to be very helpful to the ever-growing number of labs performing scRNA-seq experiments, especially in correcting for sources of technical error.

While the authors make a convincing argument for sources of a dissociation-specific transcriptional response in their data, they miss an opportunity to deliver on the goal of providing a general approach for "computational removal of transcriptional perturbation response." In the current version of the manuscript, it appears as though every new sample would require a similar experiment to identify genes activated during dissociation, as the authors fail to convey what aspects of their work can be generalized. To maximize the utility of the nice experimental data provided in the manuscript, it would be ideal if the authors could offer more concrete tools, or at least advice, for readers who want to remove this erroneous signal from their own data.

Major points

Since some of the genes you've identified as dissociated-specific can also have cell type- or time-specific expression patterns during development. Therefore, your current approach of simply removing genes seems too blunt an instrument. One way to combat this problem is to focus on the group of genes that define the dissociation response, rather than penalize individual genes. I propose that you use a more quantitative approach, similar to the common approach for correcting for cell cycle effects (see Regressing out biological effects section in Luecken and Theis, MSB, 2019). In short, you would generate a signature score using genes associated with your dissociation response (those with high fractions T-C conversions, similar to the 46 genes you defined in Table S1), then use a linear regression against this dissociation signature to correct your transcript counts. The most relevant place to test this approach would be in the data underlying Figure 2c/2d, where the correction should similarly disperse the 'activated microglia' cells without removing any genes from the dataset. This dissociation signature could easily be computed for a new dataset without additional metabolic labeling experiments, which would add significant value to the work.

Line 121 - "None of the other cell populations had a considerable portion of stress or death related GO terms (Table S3)."

This is worth further comment. In the first section of the paper, you concluded that cell stress response was the primary transcriptional signal associated with dissociation. Here, stress seems to represent a small minority of the signal in your most sensitive cell type (microglia), and is fully absent in other cell types. It seems worth your time (and indeed, for others to benefit from your results) to confront what your experiments suggest is the 'signature' of dissociation response, even if it includes unexpected genes.

"In summary, our analysis revealed that the magnitude as well as the transcriptional profile of cellular dissociation response depend strongly on sample and cell type."

I feel this summary undermines that value of your work. I was excited when I read in your abstract that you intended to aid in "computational removal of transcriptional perturbation response." As the paper went on, I was satisfied to see that you had indeed convincingly identified many genes associated with the dissociation response. However, as the paper concludes, we find these responses may be so heterogeneous that we cannot expect consistency in different cell types or even among sample replicates. If this is really the case, what hope is there for 'computational removal'? This conclusion instead suggests a blanket requirement for metabolic labeling experiments to identify and remove transcripts associated with dissociation in all samples.

Please offer some concrete advice to those hoping to correct for the dissociation effect; if your dissociation response varies greatly with sample, then it is likely already captured by standard computational approaches for batch correction in scRNA-seq data! Your experiments provide empirical evidence for the genes comprising the signature of this response, but I don't think the full potential of your findings are realized.

Minor points

In Figure 1b, shouldn't I be seeing a relative increase in in T->C substitutions over a control?

Distinguishing the different points in Figure 1b is challenging; can you separate/jitter the x-axis so the concentrations are plotted separately?

Line 86 / Figure S2 - "When comparing two independent biological replicates, we noticed that, in addition to a shared set of labeled genes, the two heart samples also showed distinct sample specific transcriptional profiles."

Please add the quantitative details here - how many and what proportion of genes were shared.

Line 91 - "This suggests that minor differences during the dissociation procedure can result in qualitatively different perturbation responses, which may potentially manifest as batch effects in single-cell RNA-seq datasets."

Can you clarify what a 'minor difference' would be here? Shouldn't the biological replicates look similar, as they were treated similarly during dissociation?

Line 95 - "might exhibit a more reproducible stress response compared to adult cardiomyocytes. Indeed, principal component analysis of labeled transcripts showed a high degree of similarity among the prenatal samples (Figure 1g, Table S2)."

Maybe reproducible is not the right word here, as the distinction is based on an n = 2 comparison for the adult samples.

Line 119 - "and observed 9.4% of the GO terms in activated microglia to be related to stress or cell death. For non-activated microglia 6.7% fell into this category."

Can you report a more meaningful number here? Fraction of GO terms isn't very useful, since the categories are not uniform in number across the ontology (some categories have many more terms than others). I would find it more useful to report the fraction of genes in each cell type that belong to stress or cell death related GO terms.

Line 84 - "Importantly, we detected no change of cardiomyocyte gene expression upon addition of 4sU during the dissociation procedure (Figure S2)."

Can you cite a specific panel here? I am not seeing this result clearly.

Figure S2b, S2c legend does not indicate the model used to generate the blue lines.

We would like to thank the reviewers for their constructive feedback, which allowed us to considerably improve the manuscript. As discussed in more detail in our point-by-point response below, we performed the following major new experiments and analysis steps:

- In order to systematically study the cell type dependence of dissociation response, we added a set of single-cell zebrafish experiments (Fig. 3).
- In order to quantify sample-to-sample variation, we increased the number of replicates for the adult cardiomyocyte analysis from 2 to 5 (Fig. 2).
- For zebrafish single cells as well as bulk cardiomyocytes, we computed a core dissociation response, which we contrast to the cell type dependent and sample-specific response.
- We now compared dissociation at 37°C to cold dissociation at 4°C (Fig. 1e) and found overall lower labeling at 4°C. However, we also detected a remaining dissociation response (including heatshock genes) as well as a small set of cold dissociation specific genes.
- We now provide more concrete advice for readers. Specifically, we now added a step-by-step protocol as a supplemental file, and we expanded the Discussion with considerations on use cases and limitations.

Reviewer #1:

Analyzing tissue composition at the single-cell resolution is of paramount importance. While spatial transcriptomics is growing up, the sensitivity in capturing RNA molecules, the spatial resolution and the extent of area that can be analysed remain limited. Instead, tissue dissociation followed by single-cell RNA-seq remains the method of choice and the sensitivity of current single-cell RNA-seq protocols is very good as thousands of transcripts across thousands of cells can be captured. However many publications have so far demonstrated that tissue dissociation leads to the activation of stress genes (see for example PMID: 32487174) but yet no generic method exists to precisely measure dissociation artifacts.

The study proposed by Neuschulz et al. proposes to fill this gap by using RNA metabolic labeling to specifically label the genes induced during tissue dissociation. Briefly, tissue dissociation is done along with 4sU (4-thiouridine) RNA metabolic labeling to mark specifically the genes that are transcribed during the procedure. The authors have demonstrated that the procedure is working on zebrafish larvae (Fig 1c,d), mouse cardiomyocytes and mouse hippocampus. The approach is fully compatible with single-cell SLAM-seq allowing to track the dissociation effects at the single-cell level (Fig.2). The authors could show (Fig 1f) that the 'same' dissociation protocol of applied to biological replicates leads to different responses highlighting again the need to implement a robust method to estimate tissue dissociation artifacts. The manuscript is very clear and the experiments are very well conducted. The manuscript will reach a very large audience.

We thank the reviewer for this positive assessment of our work.

I have a few points that I would like the authors to address mainly in the discussion and suppl. material:

1) For the next step, it is very important to make this study a generic study to be implemented in

any lab including a lab with poor results that never worked with SLAM-seq. If the author can add a step-by-step protocol it would be very important.

This is a good suggestion. We have added a step-by-step protocol as a supplemental file. Furthermore, we have expanded the Discussion in order to give more advice to potential users of the approach.

2) How methanol fixation affects the procedure? Does it lead to RNA leakage?

The reviewer raises an important point: Methanol fixation of cells is likely to lead to some degree of RNA leakage and/or may affect the detected expression profiles. However, RNA leakage seems to be relatively minor in the systems we investigated so far: In the revised manuscript, we added a new main figure with single-cell analysis of dissociated zebrafish larvae (48 hpf), a system with which we have extensive experience. In the methanol-fixed cells after scSLAM-seq we detect 800-900 genes, which is similar to fresh samples. This is in line with recent literature suggesting relatively minor effects of methanol fixation (https://doi.org/10.1186/s12864-021-07744-6), which are probably only problematic in full length sequencing and don't affect 3' based protocols much.

Furthermore, while fixation is required to make the RNA labeling protocol compatible with 10x genomics scRNA-seq, no fixation step is required for bulk analysis (where the iodoacetamide treatment can be done on extracted RNA). We also wish to note that fixation-free scSLAM-seq is possible with other experimental approaches, in particular SMART-seq (Hendriks et al., 2019; Erhard et al., 2019), drop-seq (Qiu et al., 2020, https://www.nature.com/articles/s41592-020-0935-4) and the Singleron Dynascope kit. Hence, if necessary, the fixation step can be omitted for cells where methanol treatment has a detrimental effect on data quality. We now discuss this in the text.

3) The concentration of 4sU used is very high (10 mM) (Fig 1b). We usually use concentrations in the range of hundreds of uM. Can the author explain this concentration?

Indeed, the concentration of 4sU that we used here is higher than usual. Using such a high concentration in the buffer solution has the advantage that we may reach sufficiently high intracellular 4sU concentrations faster, which is critical because of 1) our relatively short labeling time and 2) the fact that, in the initial stages of the dissociation process, we still have multi-layered tissue pieces, which take up 4sU less efficiently. That said, we would not recommend such high concentrations for applications other than measuring dissociation response, since it is not unlikely that very high 4sU concentrations may inhibit RNA metabolism upon longer exposure (PMID: 24025460). In general, we recommend checking the effect of 4sU on transcription for any RNA labeling experiment. In the revised manuscript, we investigated this for adult mouse cardiomyocytes, which require particularly long dissociation. We found that addition of 4sU during dissociation did not lead to detectable gene expression changes compared to dissociation without 4sU, suggesting that the 4sU treatment does not create gene expression artefacts (Figure S2b,c). We now explain this reasoning in the manuscript.

4) How does the 'dissociation-specific genes' found here are comparable to genes found in other reports?

In general, we found many genes that are well known to be involved in dissociation response, such as *fos/jun* and heatshock genes. We now also compared the dissociation response genes that we found in adult mouse cardiomyocytes to the top 40 dissociation response genes

described in O'Flanaghan et al., Genome Biology, 2019 (doi: 10.1186/s13059-019-1830-0) for a tumor sample. The overlap consists of *fos/jun, atf3* and *dnajb1* as well as a shared heatshock response (although the detected set of heat shock genes differs). Hence, while there is a core dissociation response, a large fraction of the detected genes are sample-specific. While the low overlap could also be partially caused methodological differences, this finding suggests that dissociation response has a large cell type-specific component.

Inspired by the reviewers' comments, we addressed the cell type dependence of dissociation response as well as sample-to-sample variation more systematically by adding a new figure (Fig. 3) on single-cell analysis of dissociation response in zebrafish larvae and by increasing the number of replicates for the adult cardiomyocyte analysis from 2 to 5 (Fig. 2). As described in the main text in more detail, we find a shared core dissociation response as well as cell type and sample dependent differences.

5) Does cold dissociation lead to dissociation artifacts? If the authors can add an experiment it would be great.

We thank the reviewer for the great suggestion. We now added an experiment in which we performed cold dissociation of zebrafish larvae (Fig. 1e). We found that overall labeling was reduced in the cold dissociation sample, which might reflect milder dissociation conditions as well as overall lower transcriptional activity of the cells at low temperature. While many of the genes detected as labeled upon dissociation at 37°C disappeared, we also found a set of remaining genes (including heatshock gene) as well as a small 4°C specific response.

Minor comments:
6) It would be informative if the authors can add systematically the number of genes that are tissue dissociation specific. Example: line 71 instead of writing 'a smaller number of genes', it would be preferable to give the numbers.

We have now added the number of genes in the figures, and we also explicitly mention those numbers in the main text wherever appropriate.

7) Fig 1g: it would be important to add in the legend what genes were used to calculate the PCA.

We now specify in the figure legend that the PCA was carried out on T to C rates of all genes with a T to C rate at least one standard deviation over the mean (in order to reduce random noise) in at least one of the adult or prenatal samples

Reviewer #2:

Review: MSB-2022-11147
A single-cell RNA labeling strategy for measuring stress response upon tissue dissociation

Summary

The authors provide a general strategy for isolating the effects of tissue dissociation on transcript abundances. Taking advantage of metabolic labeling techniques that identify newly synthesized transcripts, the authors focus on quantifying a potential source of technical variation

in single-cell RNA-seq datasets. The results in this work ought to be very helpful to the ever-growing number of labs performing scRNA-seq experiments, especially in correcting for sources of technical error.

While the authors make a convincing argument for sources of a dissociation-specific transcriptional response in their data, they miss an opportunity to deliver on the goal of providing a general approach for "computational removal of transcriptional perturbation response." In the current version of the manuscript, it appears as though every new sample would require a similar experiment to identify genes activated during dissociation, as the authors fail to convey what aspects of their work can be generalized. To maximize the utility of the nice experimental data provided in the manuscript, it would be ideal if the authors could offer more concrete tools, or at least advice, for readers who want to remove this erroneous signal from their own data.

We thank the reviewer for the positive assessment of our work and for the constructive criticism. Our main goal in this study was to develop a method that can be used for potentially problematic samples on a case-by-case basis, where it is important to validate that the dissociation procedure is not too harsh (e.g. cardiomyocytes) or to identify specific cellular responses to dissociation (e.g. microglia). However, we agree with the reviewer that it would be equally important to identify a core dissociation response program, which can then be computationally removed, potentially without further SLAM-seq experiments. As described in more detail below, we now provide additional experiments and analysis to address the reviewer's suggestion, and we also discuss the limitations of generalizing the approach.

Major points

1) Since some of the genes you've identified as dissociated-specific can also have cell type- or time-specific expression patterns during development. Therefore, your current approach of simply removing genes seems too blunt an instrument. One way to combat this problem is to focus on the group of genes that define the dissociation response, rather than penalize individual genes. I propose that you use a more quantitative approach, similar to the common approach for correcting for cell cycle effects (see Regressing out biological effects section in Luecken and Theis, MSB, 2019). In short, you would generate a signature score using genes associated with your dissociation response (those with high fractions T-C conversions, similar to the 46 genes you defined in Table S1), then use a linear regression against this dissociation signature to correct your transcript counts. The most relevant place to test this approach would be in the data underlying Figure 2c/2d, where the correction should similarly disperse the 'activated microglia' cells without removing any genes from the dataset. This dissociation signature could easily be computed for a new dataset without additional metabolic labeling experiments, which would add significant value to the work.

We thank the reviewer for this important comment. We agree that complete removal of dissociation-related genes is too simplistic and may lead to loss of potentially important information. We wish to note that, in our analysis of the mouse hippocampus in Fig. 2 (now: Fig. 4), we only removed the labeled transcripts, in order to approximate the expression of the affected genes to pre-dissociation levels. While this approach also has its limitations, it does not involve removal of genes. We now describe the procedure in more detail in the manuscript.

While we agree with the reviewer that it would in many cases be useful to regress out dissociation effects, we believe that the example of the hippocampus also highlights the limitations of such an approach: In response to stress, microglia (and macrophages in general) can undergo an activation response. Here, we showed that microglia activation was an artefact

caused by dissociation. However, in other settings (for instance in a disease context), the same microglia activation profile may be real. Regressing out this signature would therefore also remove a *bona fide* activation response. Whether this is acceptable will depend on the biological question. We now discuss this in the main text.

In order to address the reviewer's question directly, we now added an additional set of experiments, which we then used to systematically analyze cell type dependent differences in dissociation response and to test different computational approaches for removal of dissociation response. For this analysis, we chose to analyze the dissociation response of zebrafish larvae (48 hpf) at single cell resolution (Fig. 3), and we decided to compare three conditions for preparation of a single-cell suspension: 30 min dissociation; 30 min dissociation followed by 30 min of FACS (which we here used to sort for cells of expected size and granularity); and finally 30 min dissociation and 30 min FACS, followed by 30 min on ice (to simulate waiting times that arise when preparing multiple samples). In total we sequenced ~25200 cells, which computationally clustered into 16 cell types (Figure 3a).

Analysis of cell type and sample dependent differences in dissociation response

Comparing the three dissociation conditions, we observed overall low batch effects, with the exception of neuronal cells, which appeared to be more strongly affected by the FACS procedure (Figure 3b). Furthermore, muscle cells were depleted in the FACSed samples, which suggests that these cells were lost, possibly due to their larger size (Figure 3b). Analysis of T-to-C rates showed higher labeling of the FACSed samples, in line with their longer exposure to 4sU as well as differences in labeling rate between the detected cell types (Figure 3c). Comparison of the most highly labeled genes (> 10% of UMIs with >1 labeling event) revealed a shared dissociation program across cell types as well as cell type specific programs (Figure 3d, Table S4).

Computational removal of dissociation response

We decided to compare three approaches: i) Removal of only labeled transcripts (as in the hippocampus analysis in Fig. 4), ii) complete removal of the genes that are part of the core dissociation response, and iii) regressing out the core dissociation response program through linear regression (when scaling the data in Seurat). We found that none of these approaches led to noticeable changes in cluster composition (Fig. R1), which is not unexpected since larval samples can be dissociated fast with relatively mild protocols. However, we also noticed that the strong batch effect of neurons between FACSed and non-FACSed samples could not be removed by accounting for dissociation response. Upon closer examination we noticed that neurons in the FACSed samples had considerably lower fractions of mitochondrial reads compared to the non-FACSed sample. A high fraction of mitochondrial reads is often considered as a sign for damaged cells that may have lost a lot of their cytosolic mRNA due to leakage, leading to a relative increase in mitochondrial RNA. Our hypothesis is that such damaged cells may have been discarded by sorting for granularity or may have been damaged further in FACS. We believe this is a good example for a level of batch effect that is not captured by recording dissociation response.

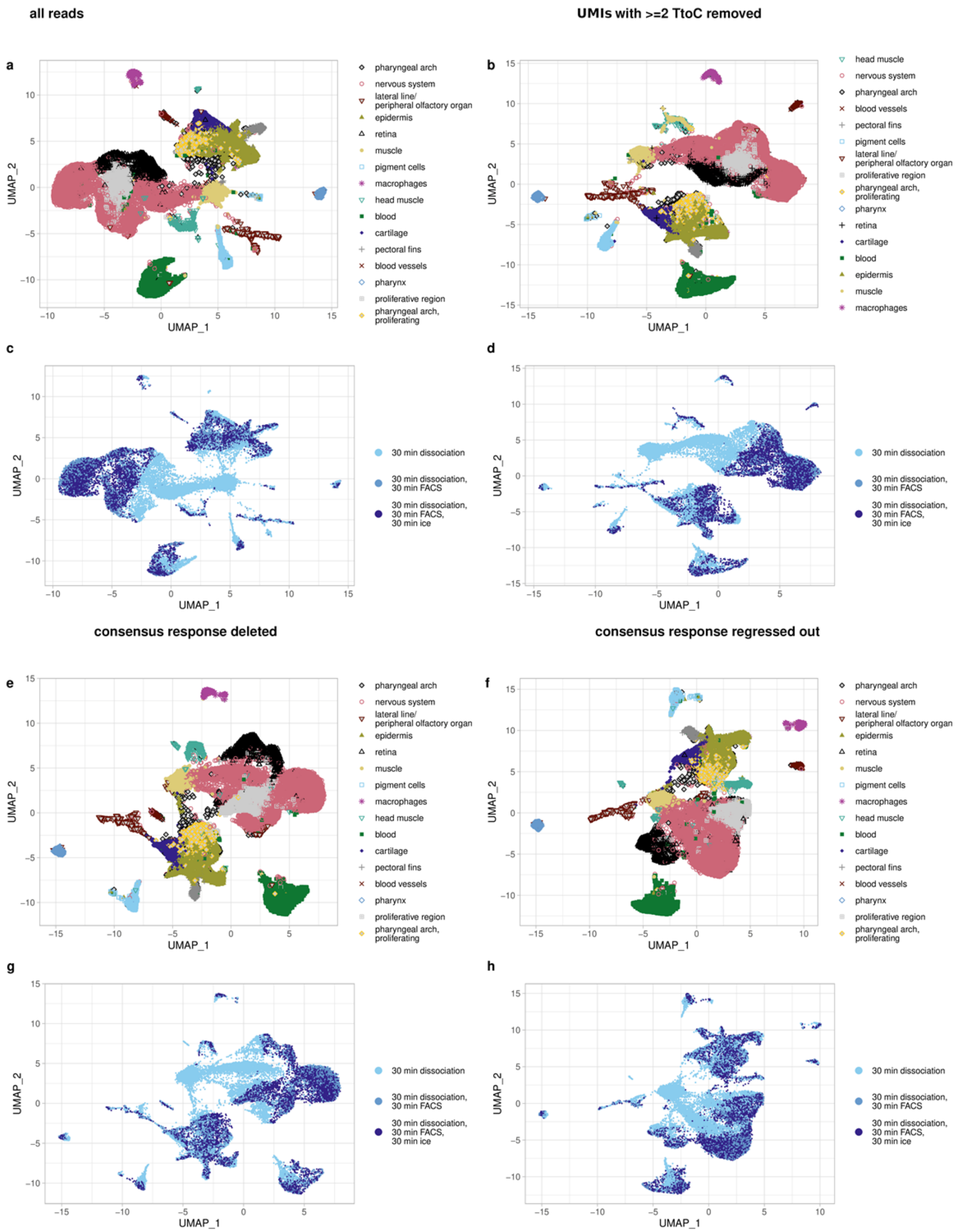

Figure R1: Removal of dissociation response through different means.
a + c) UMAP showing cell types and sample origin with all reads present.
b + d) UMIs with >=2 labeling events removed (similar to Fig. 4d)
e + g) genes constituting a consensus response (10% of reads labelled 2x in at least 10 cell types) deleted from the gene expression matrix
f + h) consensus response regressed out while scaling the data in Seurat

2) Line 121 - "None of the other cell populations had a considerable portion of stress or death related GO terms (Table S3)."

This is worth further comment. In the first section of the paper, you concluded that cell stress response was the primary transcriptional signal associated with dissociation. Here, stress seems to represent a small minority of the signal in your most sensitive cell type (microglia), and is fully absent in other cell types. It seems worth your time (and indeed, for others to benefit from your results) to confront what your experiments suggest is the 'signature' of dissociation response, even if it includes unexpected genes.

We thank the reviewer for this valuable comment, and we agree that this observation warrants further explanation. We now describe in more detail in the manuscript that the protocol for hippocampus dissociation is very mild and has been optimized specifically for gentle dissociation of brain tissue. It is hence not surprising that the overall dissociation response of the hippocampus is weaker than for e.g. cardiomyoctes (Fig. 2), which require a very harsh protocol. However, we think it is an important observation that microglia can become activated by dissociation even when using an optimized protocol, and we believe this is an example that highlights the power of our approach. In contrast to the microglia, which are very sensitive towards any type of tissue damage, the other cell types of the brain exhibit much lower dissociation response (in line with the mild dissociation protocol). We wish to note that the stress response of the microglia is in fact considerable, with ~90% of the labeled genes (≥3 transcripts with ≥2 labeling events) contributing to stress/death related GO terms in activated microglia. For non-activated microglia ~45% fell into this category.

3) "In summary, our analysis revealed that the magnitude as well as the transcriptional profile of cellular dissociation response depend strongly on sample and cell type."

I feel this summary undermines that value of your work. I was excited when I read in your abstract that you intended to aid in "computational removal of transcriptional perturbation response." As the paper went on, I was satisfied to see that you had indeed convincingly identified many genes associated with the dissociation response. However, as the paper concludes, we find these responses may be so heterogeneous that we cannot expect consistency in different cell types or even among sample replicates. If this is really the case, what hope is there for 'computational removal'? This conclusion instead suggests a blanket requirement for metabolic labeling experiments to identify and remove transcripts associated with dissociation in all samples.

We agree with the reviewer that this summary was lacking accuracy, and we have rephrased and expanded the Discussion. The reviewer raises the important question of a "core" dissociation response. We now added additional datasets: We increased the number of adult cardiomyocyte replicates from 2 to 5, and we added a new set of 3 zebrafish single-cell RNA-seq experiments. This allowed us to identify the shared dissociation response between individual cardiomyocyte samples (Fig. 2) and between different cell types in zebrafish larvae (Fig. 3), as well as sample and cell type dependent components. From this analysis it becomes clear that a shared set of core dissociation response genes exists, next to sample and cell type specific genes. Since it is difficult to draw an exact line between "core response" and "specific response" genes, we now provide detailed information of labeled genes in individual samples and cell types (cardiomyocytes: Fig. 2d, Table S3; zebrafish: Fig. 3d, Table S4; hippocampus: Fig. 4f). While it may indeed make sense to regress out the effect of core dissociation response

genes (taking into account the limitations described in our reply to question 1), we think it is equally important to be aware of the possibility of more specific effects.

4) Please offer some concrete advice to those hoping to correct for the dissociation effect; if your dissociation response varies greatly with sample, then it is likely already captured by standard computational approaches for batch correction in scRNA-seq data! Your experiments provide empirical evidence for the genes comprising the signature of this response, but I don't think the full potential of your findings are realized.

Throughout the manuscript we now distinguish core dissociation response (which can potentially be removed computationally without further SLAM-seq experiments) and sample/cell type specific response. We agree with the reviewer that the sample specific response may be removed by batch correction, but this would not deal with shared core response or cell type specific effects.

In the revised manuscript, we have taken the following steps to give more concrete advice: We included a step-by-step protocol for our approach as a supplemental file, we provide the lists of labeled genes in individual samples and cell types as supplemental tables, and we expanded the Discussion. We now explicitly state that we recommend SLAM-seq for potentially problematic samples, either because harsh dissociation conditions are required (e.g. cardiomyocytes) or because the cells are particularly responsive to perturbation (e.g. microglia). We also highlight that it is possible to regress out dissociation response from scRNA-seq datasets based on our list of core dissociation genes without performing additional SLAM-seq experiments, akin to removal of cell cycle effects in scRNA-seq, but we also note that this can be potentially problematic, since cellular stress response may not only be due to dissociation but can also be caused by biological factors (e.g. in microglia activation). In contrast to dissociation response, the cell cycle is well defined gene expression program that is strongly separated from other cellular programs (Schwabe et al, MSB, 2020), hence no such concerns apply to computational removal of cell cycle effects. In summary, we believe it is a strength of our manuscript that it supports both in-depth *de novo* experimental characterization of dissociation response as well as identification and/or computational removal of dissociation response genes according to our lists of labeled genes.

Minor points

5) In Figure 1b, shouldn't I be seeing a relative increase in in T->C substitutions over a control?

We now added an unlabeled control to the plot, showing that there is an increase in T to C substitutions in all labeled conditions.

6) Distinguishing the different points in Figure 1b is challenging; can you separate/jitter the x-axis so the concentrations are plotted separately?

We now separated the data points horizontally to improve readability.

7) Line 86 / Figure S2 - "When comparing two independent biological replicates, we noticed that, in addition to a shared set of labeled genes, the two heart samples also showed distinct sample specific transcriptional profiles."

Please add the quantitative details here - how many and what proportion of genes were shared.

We now added gene counts to the figure, as well as details in the main text.

8) Line 91 - "This suggests that minor differences during the dissociation procedure can result in qualitatively different perturbation responses, which may potentially manifest as batch effects in single-cell RNA-seq datasets."

Can you clarify what a 'minor difference' would be here? Shouldn't the biological replicates look similar, as they were treated similarly during dissociation?

While it is true that we aim for similar treatment of all samples, we are working in a living system and small discrepancies are unfortunately unavoidable, especially in a lengthy protocol. Adding more replicates of adult cardiomyocytes (our most involved dissociation protocol) led both to a consensus ‘core’ response to dissociation (Table S3) as well as to identification of sample specific genes (Fig. 2b-d).

Inspired by the reviewer's comment, we now also included additional samples in which we intentionally modified the dissociation protocol slightly by either changing the temperature (Fig. 2 (sample adult 5)) or the incubation time on ice (Fig. 3), which both affected active transcription. This underlines the importance of identifying potentially problematic genes to be aware of in downstream analysis.

9) Line 95 - "might exhibit a more reproducible stress response compared to adult cardiomyocytes. Indeed, principal component analysis of labeled transcripts showed a high degree of similarity among the prenatal samples (Figure 1g, Table S2)."

Maybe reproducible is not the right word here, as the distinction is based on an n = 2 comparison for the adult samples.

We now added more replicates (n = 5) for adult cardiomyocytes, and we observed a lower degree of similarity compared to the prenatal samples (Fig. 2c).

10) Line 119 - "and observed 9.4% of the GO terms in activated microglia to be related to stress or cell death. For non-activated microglia 6.7% fell into this category."

Can you report a more meaningful number here? Fraction of GO terms isn't very useful, since the categories are not uniform in number across the ontology (some categories have many more terms than others). I would find it more useful to report the fraction of genes in each cell type that belong to stress or cell death related GO terms.

We thank the reviewer for the suggestion and agree that reporting the fraction of genes contributing to stress/death related terms is more meaningful. The text as well as tables S2 and S5 have now been updated with the numbers and the specific contributing genes respectively.

11) Line 84 - "Importantly, we detected no change of cardiomyocyte gene expression upon addition of 4sU during the dissociation procedure (Figure S2)."

Can you cite a specific panel here? I am not seeing this result clearly.

The result is shown in Fig. S2b and c. We now updated text, figure axis labels as well as the legend to make the result more clear.

12) Figure S2b, S2c legend does not indicate the model used to generate the blue lines.

We now specified in the figure legend that the blue line shows a sliding window average (smoothed conditional mean)

Thank you for sending us your revised manuscript. We have now heard back from the two reviewers who were asked to evaluate your revised study. As you will see below, both reviewers are satisfied with the modifications made and support publication. Reviewer #1 only lists some minor issues, which we would ask you to address in minor revision. We would also ask you to address a few editorial issues listed below.

Reviewer #1:

In the revision provided by Neuschulz et al, the authors have added two figures (#2 and #3) that clearly enhance the key messages of the paper. The originality of the paper lies on the use of SLAM-seq to uncover the nascent transcripts during tissue dissociation, and the multitude of models (zebrafish, mouse; heart, brain) used to uncover the diversity of such spurious signals. Overall, the study clearly demonstrates the complexity of the signal emerging from tissue dissociation protocols and will be used by many groups as a reference paper.
I only have minor comments:

- Figure 3b: the colors are too similar.
- Figure 3c: it is not clear for me why not all the cell types are ordered similarly: for example: muscle cells has one plot, immune cells (and many others) have 3 plots.
- Figure 3d: I would try a color-code with a log-scale.

Reviewer #2:

The authors have thoroughly addressed my concerns and have gone above and beyond in including several new experiments.

Additional replicate experiments for cardiomyocytes, as well as a substantial batch of additional single-cell experiments in zebrafish, highlight the importance of the work. The authors thoroughly addressed my main concern, which was providing quantitative information on the common ("core") and cell type-specific set of genes induced by dissociation; this information is helpfully provided in Table S3/S4 and Figure 2d/3d/4f. I agree with the authors that, for some experiments, the dissociation may lead to cell type-specific effects that may be related to true biological signal, but I appreciate that they were willing to put in the extra effort to identify a common dissociation signal that could be used by those in the single cell community. The addition of a SLAM-seq protocol is helpful, as were the clarifying changes to the text and figures.

Reviewer #1:

In the revision provided by Neuschulz et al, the authors have added two figures (#2 and #3) that clearly enhance the key messages of the paper. The originality of the paper lies on the use of SLAM-seq to uncover the nascent transcripts during tissue dissociation, and the multitude of models (zebrafish, mouse; heart, brain) used to uncover the diversity of such spurious signals. Overall, the study clearly demonstrates the complexity of the signal emerging from tissue dissociation protocols and will be used by many groups as a reference paper.
I only have minor comments:

We would like to thank the reviewer for the positive feedback.

- Figure 3b: the colors are too similar.

We changed the colors in Figure 3b, which are now easier to distinguish.

- Figure 3c: it is not clear for me why not all the cell types are ordered similarly: for example: muscle cells has one plot, immune cells (and many others) have 3 plots.

We now specify in the legend of Figure 3c that cell types that were not detected in all samples have less than 3 plots. For instance, muscle cells are strongly depleted by FACS and are therefore only shown for the non-FACS sample.

- Figure 3d: I would try a color-code with a log-scale.

In Fig. 3d we now tried a color code with a log scale. However, we find that this leads to a misrepresentation of the data, since genes with low labeling rates are visually amplified and become almost indistinguishable from the genes with the highest labeling rates (Fig. R1). We would therefore prefer to keep the linear color code.

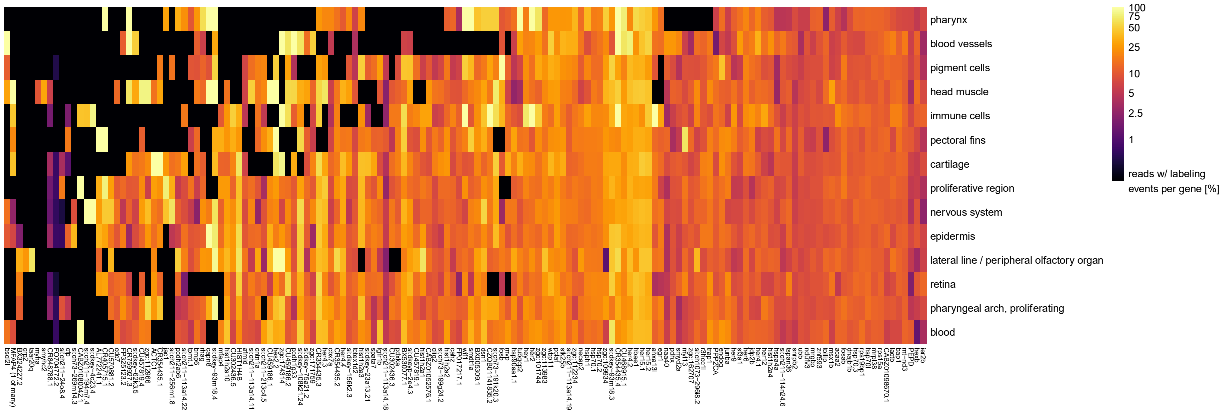

Fig R1. Version of Fig. 3d with logarithmic color code.

Reviewer #2:

The authors have thoroughly addressed my concerns and have gone above and beyond in including several new experiments.

Additional replicate experiments for cardiomyocytes, as well as a substantial batch of additional

single-cell experiments in zebrafish, highlight the importance of the work. The authors thoroughly addressed my main concern, which was providing quantitative information on the common ("core") and cell type-specific set of genes induced by dissociation; this information is helpfully provided in Table S3/S4 and Figure 2d/3d/4f. I agree with the authors that, for some experiments, the dissociation may lead to cell type-specific effects that may be related to true biological signal, but I appreciate that they were willing to put in the extra effort to identify a common dissociation signal that could be used by those in the single cell community. The addition of a SLAM-seq protocol is helpful, as were the clarifying changes to the text and figures.

We would like to thank the reviewer for the positive feedback.

2nd Revision - Editorial Decision 13th Dec 2022

Thank you again for sending us your revised manuscript. We are now satisfied with the modifications made and I am pleased to inform you that your paper has been accepted for publication.

EMBO Press Author Checklist

Corresponding Author Name: Jan Philipp Junker

Journal Submitted to: Molecular Systems Biology
Manuscript Number: MSB-2022-11147

USEFUL LINKS FOR COMPLETING THIS FORM
The EMBO Journal - Author Guidelines
EMBO Reports - Author Guidelines
Molecular Systems Biology - Author Guidelines
EMBO Molecular Medicine - Author Guidelines

Reporting Checklist for Life Science Articles (updated January
This checklist is adapted from Materials Design Analysis Reporting (MDAR) Checklist for Authors. MDAR establishes a minimum set of requirements in transparent reporting in the life sciences (see Statement of Task: 10.31222/osf.io/9sm4x). Please follow the journal's guidelines in preparing your
Please note that a copy of this checklist will be published alongside your article.

Abridged guidelines for figures
1. Data
The data shown in figures should satisfy the following conditions:
- the data were obtained and processed according to the field's best practice and are presented to reflect the results of the experiments in an accurate and unbiased manner.
- ideally, figure panels should include only measurements that are directly comparable to each other and obtained with the same assay.
- plots include clearly labeled error bars for independent experiments and sample sizes. Unless justified, error bars should not be shown for technical
- if n<5, the individual data points from each experiment should be plotted. Any statistical test employed should be justified.
- Source Data should be included to report the data underlying figures according to the guidelines set out in the authorship guidelines on Data

2. Captions
Each figure caption should contain the following information, for each panel where they are relevant:
- a specification of the experimental system investigated (eg cell line, species name).
- the assay(s) and method(s) used to carry out the reported observations and measurements.
- an explicit mention of the biological and chemical entity(ies) that are being measured.
- an explicit mention of the biological and chemical entity(ies) that are altered/varied/perturbed in a controlled manner.
- the exact sample size (n) for each experimental group/condition, given as a number, not a range;
- a description of the sample collection allowing the reader to understand whether the samples represent technical or biological replicates (including how many animals, litters, cultures, etc.).
- a statement of how many times the experiment shown was independently replicated in the laboratory.
- definitions of statistical methods and measures:
  - common tests, such as t-test (please specify whether paired vs. unpaired), simple χ2 tests, Wilcoxon and Mann-Whitney tests, can be unambiguously identified by name only, but more complex techniques should be described in the methods section;
  - are tests one-sided or two-sided?
  - are there adjustments for multiple comparisons?
  - exact statistical test results, e.g., P values = x but not P values < x;
  - definition of 'center values' as median or average;
  - definition of error bars as s.d. or s.e.m.

Please complete ALL of the questions below.
Select "Not Applicable" only when the requested information is not relevant for your study.

Materials

Newly Created Materials	Information included in the manuscript?	In which section is the information available? (Reagents and Tools Table, Materials and Methods, Figures, Data Availability Section)
New materials and reagents need to be available; do any restrictions apply?	Not Applicable	

Antibodies	Information included in the manuscript?	In which section is the information available? (Reagents and Tools Table, Materials and Methods, Figures, Data Availability Section)
For **antibodies** provide the following information:<br>- Commercial antibodies: RRID (if possible) or supplier name, catalogue number and or/clone number<br>- Non-commercial: RRID or citation	Yes	Reagents table, Methods section

DNA and RNA sequences	Information included in the manuscript?	In which section is the information available? (Reagents and Tools Table, Materials and Methods, Figures, Data Availability Section)
**Short novel DNA or RNA including primers, probes**: provide the sequences.	Not Applicable	

Cell materials	Information included in the manuscript?	In which section is the information available? (Reagents and Tools Table, Materials and Methods, Figures, Data Availability Section)
**Cell lines:** Provide species information, strain. Provide accession number in repository **OR** supplier name, catalog number, clone number, and/**OR** RRID.	Yes	
**Primary cultures:** Provide species, strain, sex of origin, genetic modification status.	Not Applicable	
Report if the cell lines were recently **authenticated** (e.g., by STR profiling) and tested for mycoplasma contamination.	Yes	Methods section

Experimental animals	Information included in the manuscript?	In which section is the information available? (Reagents and Tools Table, Materials and Methods, Figures, Data Availability Section)
**Laboratory animals or Model organisms:** Provide species, strain, sex, age, genetic modification status. Provide accession number in repository **OR** supplier name, catalog number, clone number, **OR** RRID.	Yes	Reagents table, Methods section
**Animal observed in or captured from the field:** Provide species, sex, and age where possible.	Not Applicable	
Please detail **housing and husbandry conditions**.	Yes	Methods section

Plants and microbes	Information included in the manuscript?	In which section is the information available? (Reagents and Tools Table, Materials and Methods, Figures, Data Availability Section)
**Plants:** provide species and strain, ecotype and cultivar where relevant, unique accession number if available, and source (including location for collected wild specimens).	Not Applicable	
**Microbes:** provide species and strain, unique accession number if available, and source.	Not Applicable	

Human research participants	Information included in the manuscript?	In which section is the information available? (Reagents and Tools Table, Materials and Methods, Figures, Data Availability Section)
If collected and within the bounds of privacy constraints report on age, sex and gender or ethnicity for all study participants.	Not Applicable	

Core facilities	Information included in the manuscript?	In which section is the information available? (Reagents and Tools Table, Materials and Methods, Figures, Data Availability Section)
If your work benefited from core facilities, was their service mentioned in the acknowledgments section?	Yes	

Design

Study protocol	Information included in the manuscript?	In which section is the information available? (Reagents and Tools Table, Materials and Methods, Figures, Data Availability Section)

If study protocol has been **pre-registered, provide DOI in the manuscript**. For clinical trials, provide the trial registration number **OR** cite DOI.	Not Applicable	
Report the **clinical trial registration number** (at ClinicalTrials.gov or equivalent), where applicable.	Not Applicable	

Laboratory protocol	Information included in the manuscript?	In which section is the information available? (Reagents and Tools Table, Materials and Methods, Figures, Data Availability Section)
Provide DOI OR other citation details if **external detailed step-by-step protocols** are available.	Yes	Methods section

Experimental study design and statistics	Information included in the manuscript?	In which section is the information available? (Reagents and Tools Table, Materials and Methods, Figures, Data Availability Section)
Include a statement about **sample size** estimate even if no statistical methods were used.	Yes	Methods section, Figures
Were any steps taken to minimize the effects of subjective bias when allocating animals/samples to treatment (e.g. **randomization procedure**)? If yes, have they been described?	Not Applicable	
Include a statement about **blinding** even if no blinding was done.	Yes	Methods section
Describe **inclusion/exclusion criteria** if samples or animals were excluded from the analysis. Were the criteria pre-established?<br><br>If sample or data points were omitted from analysis, report if this was due to attrition or intentional exclusion and provide justification.	Not Applicable	
For every figure, are **statistical tests** justified as appropriate? Do the data meet the assumptions of the tests (e.g., normal distribution)? Describe any methods used to assess it. Is there an estimate of variation within each group of data? Is the variance similar between the groups that are being statistically compared?	Yes	Methods section, Figures

Sample definition and in-laboratory replication	Information included in the manuscript?	In which section is the information available? (Reagents and Tools Table, Materials and Methods, Figures, Data Availability Section)
In the figure legends: state number of times the experiment was **replicated** in laboratory.	Yes	
In the figure legends: define whether data describe **technical or biological replicates**.	Yes	

Ethics

Ethics	Information included in the manuscript?	In which section is the information available? (Reagents and Tools Table, Materials and Methods, Figures, Data Availability Section)
Studies involving **human participants**: State details of **authority granting ethics approval** (IRB or equivalent committee(s), provide reference number for approval.	Not Applicable	
Studies involving **human participants**: Include a statement confirming that **informed consent** was obtained from all subjects and that the experiments conformed to the principles set out in the WMA Declaration of Helsinki and the Department of Health and Human Services Belmont Report.	Not Applicable	
Studies involving **human participants:** For publication of **patient photos**, include a statement confirming that consent to publish was obtained.	Not Applicable	
Studies involving experimental **animals**: State details of **authority granting ethics approva**l (IRB or equivalent committee(s), provide reference number for approval. Include a statement of compliance with ethical regulations.	Yes	Methods section
Studies involving **specimen and field samples:** State if relevant **permits** obtained, provide details of authority approving study; if none were required, explain why.	Not Applicable	

Dual Use Research of Concern (DURC)	Information included in the manuscript?	In which section is the information available? (Reagents and Tools Table, Materials and Methods, Figures, Data Availability Section)
Could your study fall under dual use research restrictions? Please check biosecurity documents and list of select agents and toxins (CDC): https://www.selectagents.gov/sat/list.htm	Not Applicable	
If you used a select agent, is the security level of the lab appropriate and reported in the manuscript?	Not Applicable	
If a study is subject to dual use research of concern regulations, is the name of the **authority granting approval and reference number** for the regulatory approval provided in the manuscript?	Not Applicable	

Reporting

The MDAR framework recommends adoption of discipline-specific guidelines, established and endorsed through community initiatives. Journals have their own policy about requiring specific guidelines and recommendations to complement MDAR.

Adherence to community standards	Information included in the manuscript?	In which section is the information available? (Reagents and Tools Table, Materials and Methods, Figures, Data Availability Section)
State if relevant guidelines or checklists (e.g., **ICMJE, MIBBI, ARRIVE, PRISMA**) have been followed or provided.	Yes	
For **tumor marker prognostic studies,** we recommend that you follow the **REMARK** reporting guidelines (see link list at top right). See author guidelines, under 'Reporting Guidelines'. Please confirm you have followed these guidelines.	Not Applicable	
For **phase II and III randomized controlled trials**, please refer to the **CONSORT** flow diagram (see link list at top right) and submit the CONSORT checklist (see link list at top right) with your submission. See author guidelines, under 'Reporting Guidelines'. Please confirm you have submitted this list.	Not Applicable	

Data Availability

Data availability	Information included in the manuscript?	In which section is the information available? (Reagents and Tools Table, Materials and Methods, Figures, Data Availability Section)
Have **primary datasets** been deposited according to the journal's guidelines (see 'Data Deposition' section) and the respective accession numbers provided in the Data Availability Section?	Yes	
Were **human clinical and genomic datasets** deposited in a public access-controlled repository in accordance to ethical obligations to the patients and to the applicable consent agreement?	Not Applicable	
Are **computational models** that are central and integral to a study available without restrictions in a machine-readable form? Were the relevant accession numbers or links provided?	Yes	
If publicly available data were reused, provide the respective **data citations in the reference list**.	Not Applicable	
